# Association algorithm to mine the rules that govern enzyme definition and to classify protein sequences

**DOI:** 10.1186/1471-2105-7-304

**Published:** 2006-06-15

**Authors:** Shih-Hau Chiu, Chien-Chi Chen, Gwo-Fang Yuan, Thy-Hou Lin

**Affiliations:** 1 Bioresource Collection and Research Center, Food Industry Research and Development Institute, HsinChu, Taiwan; 2 Institute of Molecular Medicine/Department of Life Science, National Tsing Hua University, HsinChu, Taiwan

## Abstract

**Background:**

The number of sequences compiled in many genome projects is growing exponentially, but most of them have not been characterized experimentally. An automatic annotation scheme must be in an urgent need to reduce the gap between the amount of new sequences produced and reliable functional annotation. This work proposes rules for automatically classifying the fungus genes. The approach involves elucidating the enzyme classifying rule that is hidden in UniProt protein knowledgebase and then applying it for classification. The association algorithm, *Apriori*, is utilized to mine the relationship between the enzyme class and significant InterPro entries. The candidate rules are evaluated for their classificatory capacity.

**Results:**

There were five datasets collected from the Swiss-Prot for establishing the annotation rules. These were treated as the training sets. The TrEMBL entries were treated as the testing set. A correct enzyme classification rate of 70% was obtained for the prokaryote datasets and a similar rate of about 80% was obtained for the eukaryote datasets. The fungus training dataset which lacks an enzyme class description was also used to evaluate the fungus candidate rules. A total of 88 out of 5085 test entries were matched with the fungus rule set. These were otherwise poorly annotated using their functional descriptions.

**Conclusion:**

The feasibility of using the method presented here to classify enzyme classes based on the enzyme domain rules is evident. The rules may be also employed by the protein annotators in manual annotation or implemented in an automatic annotation flowchart.

## Background

The number of sequences generated by many genome projects is soaring exponentially but most of them have not been characterized experimentally. Manual annotation methods have been proposed by experts and are popular for use at the genome centers, but their annotation capacities are exceeded by the fast growing genome data. An automatic annotation scheme is in urgent need to speed up reliable functional annotation on new sequences produced. Automatic annotation provides an efficient procedure for analyzing the gene sequences. Most automatic solutions used to characterize the gene sequences are based on a high-level sequence similarity search against some known protein databases such as using the BLAST or FASTA program. The correlation between sequence composition and functional characterization provides the foundation for transferring functional knowledge from a biochemically characterized protein to a homologous but uncharacterized one. However, sequence composition bias and database updating commonly influence the results of similarity searches, and they do not yield the exact share between biological function and domain composition based on the similarity threshold used [1]. Many annotation packages have recently been developed. For example, a basic annotation, which is directly transferred from a homologue entry using a similarity threshold, is offered by the package GeneQuiz [2]. Other packages, such as Rulebase [3] and HAMAP [1], use the classifying rules and are supported by the judgment of a curator.

In the post-genomic era, the functional annotations are of great importance in understanding the real cellular processes. A variety of enzymes and pathway databases including Ecocyc, Enzyme, and KEGG, have been built to facilitate the prediction of the metabolic pathway. Such databases are supplied as reference databases in virtual construction of the metabolic networks of other organisms. On the pathway map, enzymes are the main components used for linking the metabolic networks. The fundamental units of enzyme structure governing folding and function are domains of protein [4]. A domain is believed to be able to fold independently into a stable three-dimensional structure to perform a specific function. In general, a protein would comprise a single domain or several different domains. It is clear that the domain composition of a protein determines its function and pathways in which it participates [5]. In other words, the protein function may be inferred from the domain composition which is then used to annotate the unknown sequences sharing the same domain composition with the protein. More importantly, such rules are invariable unlike the BLAST results that typically vary as the database is updated. Many tools are available for detecting the constituents of proteins such as CDART (NCBI) and InterProScan. InterPro is a database of protein families, domains, and functional sites where identifiable characteristics of known proteins can be applied to annotate unknown protein sequences [6]. The tools of InterPro, InterProScan, can be also used to annotate the single domain protein sequence; however, it may be difficult to make a decision on the annotation of multi-domain proteins by the method.

This work proposes a machine learning method for identifying enzyme classes according to the rules that are related to the protein domain composition. Using rules generated by machine learning algorithms, Kretschmann *et al*. [7] and Bazzan *et al*. [8] have successfully annotated the Trembl database. They adopted the decision tree algorithm to obtain rules from the Swiss-Prot entries that are cross-referenced to the InterPro database. They then used these rules to assign appropriate keywords to the TrEMBL entries [7,8]. In this study, an association algorithm is used to mine the rules linking enzymes and domains and they are then used to annotate enzyme classes automatically. The association algorithm has been extensively employed to analyze market baskets. It is applied primarily to determine the relationships among items in a large dataset. In market basket analysis, large associated itemsets always represent items that are likely to be purchased together by customers in a single transaction. The association algorithm has also been employed to mine gene expression data [9] and medical data [10]. This investigation utilizes an association algorithm to mine the rules linking enzymes and domains from the Swiss-Prot protein knowledgebase. The enzyme class and InterPro accession number (henceforth IPR Acc's) are treated consistently as items in searching for rules governing the enzyme domain composition. These rules may be useful even for annotators who do not have deep knowledge on the definitions of enzyme classes.

## Results and discussion

### Data preparation

This work seeks to annotate unknown genes and establishes virtual metabolic pathways using the bioinformatics approach based on progress made in the Monascus genome project at the authors' institute. Only few Monascus genes have been biochemically characterized so far. Numerous well-characterized proteins have been stored in a public database so that it is feasible to mine the classified rules from a protein knowledgebase. The BLAST is a fast but insufficient method for annotating unknown genes because it does not provide information on the functional domain. Analyzing the constituent domains of a gene enables the determination of possible functions of the gene. However, making a decision regarding the annotation of a multi-domain protein is difficult. In this study, an annotation model was established by applying rules derived from the domain compositions in some well-characterized proteins. The concept of annotation using the domain composition was further investigated. Five datasets (Table 1) were used to mine the association rules, which were then evaluated. All the datasets used herein have the EC class and IPR description. In the preliminary investigation, all the IPR Accs of each Swiss-Prot entry were utilized to determine the association rules. Unfortunately, some IPR Accs were presented as a single rule whose entries were linked to approximate sequence position but assigned with different accession numbers. To reduce the redundant and insignificant ones such as the glycosylation site and others, the IDA in InterPro was employed to filter the IPR entries.

### Association algorithm used to mine enzyme composition

**Figure 1:**
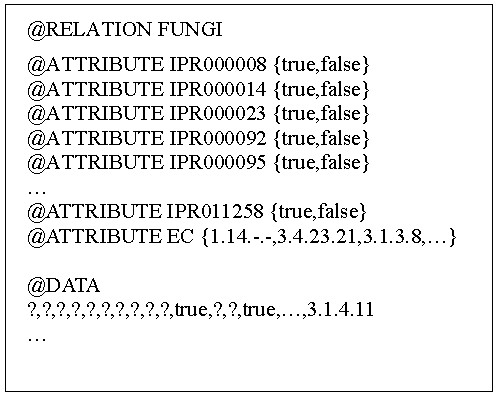
Input file to the Weka program. The false attribute was replaced with a "?" mark as a msising datum to prevent the generation of useless association rules.

Many data mining methods have been applied in the biological researches. For example, a decision tree has been used in keyword annotation in the Swiss-Prot [7] and PIGS [8] projects. Herein, the association algorithm was employed to find rules to perform automatic annotation. The association algorithm has been extensively used to elucidate the consumptive behavior. These rules are ordinarily mined from numerous transaction records. Similar to the market basket analysis, the EC class and IPR Acc's were treated as a single transaction record in every training entry. In the training file (Fig. 1), each instance was composed of all the attributes (IPR Acc's) of the training dataset and all the EC class was included in the target class. The results indicated that various rules were obtained simultaneously. The candidate association rules were found redundant and many were subsets of larger frequent itemsets. Table 2 presented the subset of fungous association rules thus obtained. The complete set of rules was shown in the additional file [see Additional file 1]. The rules revealed, for instance, when the InterProScan results of the protein sequence gave IPR000873, IPR001031, IPR001242, and IPR006163, the protein was identified as EC 6.3.2.26. Table 3 listed the association rules obtained from the five datasets. About 40 ~ 70% of the rules thus obtained were the multiple domain rules (> = 2 domains). Although the single domain rules dominate some datasets, the multiple domain rules are more important in the annotation tasks.

### Evaluation of candidate rules

As presented in Table 4, the testing dataset from TrEMBL was used to evaluate the candidate rules. The precision was around 70% for the prokaryote dataset (A and B) though the coverage was less than 50%. The precision and coverage for the eukaryote datasets (C, D and E) were better than those for the prokaryote ones. The prokaryote training dataset appears to be more diversified than the eukaryote one which results in the number of rules obtained for the former was less than that for the latter. Additionally, the prediction coverage was enhanced substantially while there were redundancies remaining in the candidate rules. In fact, the rules from the subsets of the large itemset were used to predict entries that were not exactly matched with the rules from the large itemset though the prediction accuracy was slightly decreased.

Table 5 displays the cross evaluation results for the five datasets. Both precision and confidence estimated from the cross evaluation on various phylogenetic datasets (such as using a fungus testing dataset to evaluate the plant rules) were worse than those estimated on the same taxonomic dataset. This reveals that the accuracy of the prediction depends on the taxonomic relationship between the training and testing datasets. The closer the taxonomic relationship between datasets used the greater the predictive capacity obtained. Moreover, we found that there was at least 40% accuracy in different taxonomic cross-validation. It seems that some domain compositions of enzyme were similar among different taxonomic dataset. Additionally, the prediction accuracy may reflect the taxonomic relationship in the different dataset. Yang et al. [11] proposed that using only the presence or absent of a protein domain architecture can determinate the phylogeny of 174 complete genomes. Our results also reveal that domain composition of enzyme is highly similar in the same taxonomic clade.

Furthermore, the accuracy of the presented method was compared with the rules obtained from the InterPro database. These rules were parsed where the IPR Acc's were cross-referenced to ENZYME in the entry_xref table of the InterPro database. The rules such as {IPR001711, EC 3.1.4.11} were retained for providing the enzyme identification. There were five testing datasets used to evaluate the parsed ones. As shown in Table 6, the identification accuracy was below 65%. The results revealed that it was not suitable to directly parse the cross-reference between enzyme and InterPro Acc's without classifying the dataset beforehand. In other words, as mentioned above, the identification of enzyme classes should use the closer taxonomic rules. Moreover, the rules generated from the association algorithm were highly specific in the closer taxonomic testing dataset. The association algorithm was able to select more confident rules in the protein database. As shown in Table 7 and 8, our single domain rules can identify enzyme classes with high accuracy, while multiple domain rules can lift the hit ratio in the enzyme identification. In addition, the remaining datasets which were not annotated with an EC class in the fungus dataset of Swiss-Prot entries were further employed to evaluate the fungus rule set. A total of 88 out of 5085 test entries were found to match with the fungus rule set (Table 9). Most of these were otherwise poorly annotated by their functional description. These indicate that the rules mined from the association algorithm were unique to the enzyme class and could be used to annotate some unknown protein sequences.

The precision and confidence of each EC class was also evaluated in the fungus dataset. Both quantities were varied among all the EC classes tested (data not shown here). However, a precision of greater than 75% was obtained for 60% of the EC classes tested (data not shown.). In this study, the Swiss-Prot entries were chosen as the training while the TrEMBL entries as the test set. We aimed to find the EC classifying rules that are hidden in the protein knowledgebase and to estimate the accuracy of the classifying method. The rules mined and presented here can be used by an annotator to perform manual annotation. They can be also implemented in an automatic annotation flowchart. They are also feasible to be used in identifying enzyme classes based on their IPR signature.

## Conclusion

This report proposed an alternative approach on employing the association algorithm. The association algorithm is commonly used to identify large and frequent item sets and mine hidden relationships among items. The concept can be applied in many fields other than market basket analysis. The method is extended here to mine the association rules which are then applied to identify enzyme classes. The current prediction scheme emphasizes on identifying enzymes of taxonomically closed datasets. Rule sets generated from the eukaryote training datasets can be used to assign the EC classes accurately to poorly annotated entries whose real enzyme function remain unknown. Extending the method to predict other types of data, including the transcription factors and structure proteins, is also worthwhile. However, the low coverage is a shortcoming of the presented scheme. The matching coverage depends on the quality of the training dataset which may be extended as a combination of various datasets with each being closed in taxonomic relationship. Moreover, more rules may be generated using other association algorithms except the *Apriori *one.

## Methods

### Data preparation

There were five distinctly taxonomic datasets referring to the NEWT [12] (UniProt 4.1) being downloaded (Table 1). The entries that have multiple EC description numbers were ignored. The training datasets were the Swiss-Prot entries while the test datasets were taken from TrEMBL. The EC numbers corresponding to the Swiss-Prot entries were parsed from the field 'Description' in the Swiss-Prot database. The InterPro entries that were relevant to the Swiss-Prot entries were extracted from the InterPro database (release 9.0). The extracted data were stored in a MySQL database. The IDA (InterPro Domain Architecture) definition was also extracted from the InterPro database. Not all of the InterPro entries that corresponded to the UniProt entries were treated as the training or testing attributes. The redundant and insignificant InterPro entries were removed based on the IDA definition. The redundancy-deprived data were also stored in the MySQL database.

### Appling association rules determine potential enzyme composition

The WEKA machine learning package [13] which is a freeware issued under the GNC General Public License was used to mine the association rules. The enzyme class and IPR Accs were consistently treated as items in searching for rules that governing the enzyme composition. For example, {IPR002019, IPR002026, IPR006680, IPR011612 and EC 3.5.1.5} in O00084 were considered to be a single transaction in the context of market basket analysis. The data stored in the MySQL database were transformed into the WEKA format (Fig. 1). The first line indicates which dataset was analyzed. (In this case, the file refers to the fungi training dataset.). The 822 lines (each headed with '@ATTRIBUTE IPR') that followed were all individual IPR Accs in the fungi training dataset. All the attributes were specified only by the value of 'true' or 'false' to indicate whether or not they were related with the given Swiss-Prot entry. The last attribute (labeled with '@ATTRIBUTE EC') was the classified target or class that consisted of all the unique EC classes in the training dataset. Finally, the 3666 lines that were behind the '@DATA' label were all the Swiss-Prot entries in the fungi training dataset used. Each of these comprises 823 entries and was separated by a comma. The interior 'false' value was replaced by a question mark to avoid the meaningless rules.

The *Apriori *[14,15] module in the WEKA package, implemented on a linux workstation, was employed to scan the frequent itemsets and determine the associative relationships. The association rule model represents rules where a set of items was associated with each other. For instance, a rule could specify a certain product that was frequently bought in combination with other products. The rules were extracted from some large and frequently occurring itemsets. An itemset was regarded as frequent if the possibility of its occurrence exceeded a specified minimal support criterion. The algorithm proceeds iteratively to identify the frequent itemsets consisting of a single item. Then, the identified frequent itemsets were expanded with one more item to generate larger frequent itemsets. After all the frequent itemsets were identified, the candidate rules were screened through the following 'lift' criterion.

Support (AB) = *P*(*A*+*B*)     (1)

Confidence (AB) = *P*(*B*|*A*)     (2)

lift (AB)=P(B|A)P(B)     (3)
 MathType@MTEF@5@5@+=feaafiart1ev1aaatCvAUfKttLearuWrP9MDH5MBPbIqV92AaeXatLxBI9gBaebbnrfifHhDYfgasaacH8akY=wiFfYdH8Gipec8Eeeu0xXdbba9frFj0=OqFfea0dXdd9vqai=hGuQ8kuc9pgc9s8qqaq=dirpe0xb9q8qiLsFr0=vr0=vr0dc8meaabaqaciaacaGaaeqabaqabeGadaaakeaacqqGSbaBcqqGPbqAcqqGMbGzcqqG0baDcaaMc8UaeiikaGIaeeyqaeKaeeOqaiKaeiykaKIaeyypa0ZaaSaaaeaacqWGqbaucqGGOaakcqWGcbGqcqGG8baFcqWGbbqqcqGGPaqkaeaacqWGqbaucqGGOaakcqWGcbGqcqGGPaqkaaGaaCzcaiaaxMaadaqadaqaaiabiodaZaGaayjkaiaawMcaaaaa@46A9@

where *P(B|A) *was the conditional probability of *B *given *A*, and *P(A) *or *P(B) *was the probability of *A *or *B *over all instances. The probability was defined as the observed frequency in the data set. The support of the rule was the relative frequency of transactions containing both A and B. The lift was the related measure of strength of the association. Positive correlation was indicated by lift > 1 while negative correlation was indicated by lift < 1. A large frequent itemsets were subdivided into smaller ones in numerous ways to generate the candidate association rules. The candidate association rules were redundant and many of them were subsets of larger frequent itemsets. However, the rules mined herein were of the form *{A,B,C}⇒{D} *but not *{A,B}⇒{C,D} *or *{A}⇒{B,C,D}*. For example, *{IPR000873, IPR006163, IPR010080} ⇒ {1.2.1.31}*. Because most of the support values of items were between 1 and 50, a minimum support value of 0.09% was set herein to indicate that the attribute must appear 2.7 times in 3000 instances. The threshold of confidence was 0.6 and the corresponding lift value was between 10 and 30.

### Evaluation of the candidate rules

The criterion satisfied rules were stored in the MySQL database for further evaluation. The testing dataset was used to evaluate the candidate rules governing the enzyme domain composition. Each test datum (separated by commas) was treated as a single string and matched with the set of rules (also separated by commas and treated as a single string) to find the corresponding EC class. The precision of EC class matching (testing dataset to rules set) and the confidence were evaluated using the following equations as given by Kretschmann *et al*. [7].

P=precision=TPTP+FP     (4)
 MathType@MTEF@5@5@+=feaafiart1ev1aaatCvAUfKttLearuWrP9MDH5MBPbIqV92AaeXatLxBI9gBaebbnrfifHhDYfgasaacH8akY=wiFfYdH8Gipec8Eeeu0xXdbba9frFj0=OqFfea0dXdd9vqai=hGuQ8kuc9pgc9s8qqaq=dirpe0xb9q8qiLsFr0=vr0=vr0dc8meaabaqaciaacaGaaeqabaqabeGadaaakeaacqWGqbaucqGH9aqpcqqGWbaCcqqGYbGCcqqGLbqzcqqGJbWycqqGPbqAcqqGZbWCcqqGPbqAcqqGVbWBcqqGUbGBcqGH9aqpdaWcaaqaaiabdsfaujabdcfaqbqaaiabdsfaujabdcfaqjabgUcaRiabdAeagjabdcfaqbaacaWLjaGaaCzcamaabmaabaGaeGinaqdacaGLOaGaayzkaaaaaa@47DF@

confidence=P+z22n−z∗Pn−P2n+z24n21+z2n     (5)
 MathType@MTEF@5@5@+=feaafiart1ev1aaatCvAUfKttLearuWrP9MDH5MBPbIqV92AaeXatLxBI9gBaebbnrfifHhDYfgasaacH8akY=wiFfYdH8Gipec8Eeeu0xXdbba9frFj0=OqFfea0dXdd9vqai=hGuQ8kuc9pgc9s8qqaq=dirpe0xb9q8qiLsFr0=vr0=vr0dc8meaabaqaciaacaGaaeqabaqabeGadaaakeaacqqGJbWycqqGVbWBcqqGUbGBcqqGMbGzcqqGPbqAcqqGKbazcqqGLbqzcqqGUbGBcqqGJbWycqqGLbqzcqGH9aqpdaWcaaqaaiabdcfaqjabgUcaRmaalaaabaGaemOEaO3aaWbaaSqabeaacqaIYaGmaaaakeaacqaIYaGmcqWGUbGBaaGaeyOeI0IaemOEaONaey4fIOYaaOaaaeaadaWcaaqaaiabdcfaqbqaaiabd6gaUbaacqGHsisldaWcaaqaaiabdcfaqnaaCaaaleqabaGaeGOmaidaaaGcbaGaemOBa4gaaiabgUcaRmaalaaabaGaemOEaO3aaWbaaSqabeaacqaIYaGmaaaakeaacqaI0aancqWGUbGBdaahaaWcbeqaaiabikdaYaaaaaaabeaaaOqaaiabigdaXiabgUcaRmaalaaabaGaemOEaO3aaWbaaSqabeaacqaIYaGmaaaakeaacqWGUbGBaaaaaiaaxMaacaWLjaWaaeWaaeaacqaI1aqnaiaawIcacaGLPaaaaaa@5DC5@

*n *= *TP *+ *FP *    (6)

where *TP *represents the "*T*rue *P*ositives" and *FP *represents the "*F*alse *P*ositives" and *z *is a constant, 1.96 (for 95% confidence).

## Authors' contributions

SHC implemented the computational approach, performed the analysis and drafted the manuscript. CCC and GYF participated in the design of this study. THL participated in the design of this study, interpreted the results, and wrote the manuscript. All authors read and approved the final manuscript.

## Supplementary Material

Additional File 1 All rules generated from the fungus training dataset. Click here for file

## Figures and Tables

**Table 1:** Five distinctly taxonomic datasets referring to the NEWT were used for generating and evaluating rules.

	A	B	C	D	E
Training Instances	3251	7522	3666	1791	4502
Training Attributes	657	784	823	589	551
Testing Instances	3440	10226	1759	1551	5022
Testing Attributes	777	1054	491	212	507

A: actinobacteria B: bacillales C: fungi D: nematode + arthropoda E: viridiplantae

**Table 2:** Subset of rules generated from the fungus training dataset. The complete set of rules was shown in the additional file [see Additional file 1].

Association rules	EC ID
IPR000873,IPR001031,IPR001242,IPR006163	6.3.2.26
IPR001031,IPR001242,IPR006163	6.3.2.26
IPR000873,IPR001031,IPR006163	6.3.2.26
IPR000873,IPR001031,IPR001242	6.3.2.26
IPR002314,IPR002317	6.1.1.11
IPR001926,IPR002028	4.2.1.20
IPR001031,IPR001242	6.3.2.26
IPR000873,IPR001031	6.3.2.26
IPR000850,IPR007862	2.7.4.3
IPR007862	2.7.4.3
IPR004308	6.3.2.2
IPR003171	1.5.1.20
IPR002934	2.7.7.19
IPR000873,IPR001031,IPR001242,IPR006164	6.3.2.26
IPR001031,IPR001242,IPR006164	6.3.2.26
IPR000873,IPR001031,IPR003679	6.3.2.26
IPR000873,IPR001031,IPR008600	6.3.2.26
IPR002314,IPR002318	6.1.1.12
IPR001926,IPR002029	4.2.1.21
IPR001031,IPR001243	6.3.2.26
IPR000873,IPR001032	6.3.2.26
IPR000850,IPR007863	2.7.4.3
IPR007862	2.7.4.3
IPR004308	6.3.2.3
IPR000873,IPR001031,IPR001242,IPR006164	6.3.2.26
IPR001031,IPR001242,IPR006164	6.3.2.26
IPR000873,IPR001031,IPR003679	6.3.2.26
IPR000873,IPR001031,IPR008600	6.3.2.26
IPR002314,IPR002318	6.1.1.12
IPR001926,IPR002029	4.2.1.21

**Table 3:** Number of rules and classified EC generated from the training dataset.

	A	B	C	D	E
Rules	624	607	920	1096	428
EC	254	229	167	168	153
multiple domain rule	40%	43%	69%	72%	42%

A: actinobacteria B: bacillales C: fungi D: nematode + arthropoda E: viridiplantae

**Table 4:** Evaluation of the generated candidate rules. The testing dataset was used to validate the corresponding set of rules. For instance, the fungus testing dataset was used to evaluate the set of rules generated from the fungus training dataset.

	A	B	C	D	E
precision	71%	76%	87%	88%	77%
confidence	69%	74%	85%	85%	75%
coverage*	43%	38%	60%	54%	56%

A: actinobacteria B: bacillales C: fungi D: nematode + arthropoda E: viridiplantae

*: coverage = the hit ratio of testing data

**Table 5:** Cross validation of the rule sets. The fungus testing dataset was used to evaluate the rule sets generated from the A, B, C, D, and E training datasets.

	A rule set		B rule set		C rule set		D rule set		E rule set	
	precision	confidence	precision	confidence	precision	confidence	precision	confidence	precision	confidence
A testing data	71%	69%	72%	69%	59%	56%	46%	42%	52%	48%
B testing data	68%	67%	76%	74%	61%	59%	43%	41%	51%	49%
C testing data	72%	68%	69%	65%	87%	85%	79%	76%	66%	62%
D testing data	66%	61%	43%	38%	74%	72%	88%	85%	68%	65%
E testing data	52%	50%	65%	62%	60%	58%	64%	62%	77%	75%

A: actinobacteria B: bacillales C: fungi D: nematode + arthropoda E: viridiplantae

**Table 6:** The five datasets used to evaluate the rules parsed from the InterPro database.

	the parsed rules^# ^		
	precision	Confidence	coverage*
A testing data	52%	48%	23%
B testing data	51%	50%	24%
C testing data	56%	52%	26%
D testing data	47%	41%	20%
E testing data	64%	62%	47%

A: actinobacteria B: bacillales C: fungi D: nematode + arthropoda E: viridiplantae

*: coverage = the hit ratio of testing data

^#^: The dataset was parsed from the entry xref table of the InterPro database. The IPR Acc's were corresponding to ENZYME.

**Table 7:** Accuracy of the single domain rules divided from the five rule sets.

	A	B	C	D	E
precision	68%	71%	87%	85%	77%
confidence	65%	70%	85%	82%	75%
coverage*	31%	28%	48%	41%	46%

A: actinobacteria B: bacillales C: fungi D: nematode + arthropoda E: viridiplantae

*: coverage = the hit ratio of testing data

**Table 8:** Accuracy of the multiple domain rules divided from the five rule sets.

	A	B	C	D	E
precision	79%	87%	87%	97%	76%
confidence	75%	85%	82%	93%	72%
coverage*	12%	10%	11%	13%	10%

A: actinobacteria B: bacillales C: fungi D: nematode + arthropoda E: viridiplantae

*: coverage = the hit ratio of testing data

**Table 9:** Examples of the matching entries which were not annotated with an EC class in the remaining fungus dataset of Swiss-Prot entries were predicted using the fungus rule set.

Swiss-Prot ID	Description	predicted ec	lift score
P38811	Transcription-associated protein 1 (p400 kDa component of SAGA).	2.7.1.137	523.71
P23202	URE2 protein.	2.5.1.18	523.71
Q00717	Putative sterigmatocystin biosynthesis protein stcT.	2.5.1.18	523.71
Q6BM74	URE2 protein.	2.5.1.18	523.71
Q7LLZ8	URE2 protein.	2.5.1.18	523.71
Q8NJR4	URE2 protein.	2.5.1.18	523.71
Q8NJR5	URE2 protein.	2.5.1.18	523.71
Q96WL3	URE2 protein.	2.5.1.18	523.71
Q96X43	URE2 protein.	2.5.1.18	523.71
Q96X44	URE2 protein.	2.5.1.18	523.71
P43589	Hypothetical 52.2 kDa protein in MPR1-GCN20 intergenic region.	3.1.2.15	122.2
O42908	Hypothetical protein C119.17 in chromosome II.	3.4.24.64	192.95
Q10068	Hypothetical protein C3H1.02c in chromosome I.	3.4.24.64	192.95
Q12496	Hypothetical 118.4 kDa protein in WRS1-PKH2 intergenic region.	3.4.24.64	192.95
P39994	Hypothetical 61.3 kDa protein in URA3-MMS21 intergenic region.	4.1.1.1	261.86
P43546	Hypothetical 16.6 kDa protein in THI5-AGP3 intergenic region.	1.1.1.-	112.42
P38169	Hypothetical 52.4 kDa protein in ATP1-ROX3 intergenic region precursor.	1.14.99.7	523.71
P10662	Mitochondrial 40S ribosomal protein MRP1.	1.15.1.1	107.82
P47141	Hypothetical 30.2 kDa protein in YUH1-URA8 intergenic region.	1.15.1.1	107.82
P53109	Hypothetical 65.8 kDa protein in SUT1-RCK1 intergenic region.	1.16.1.7	407.33
P36168	Hypothetical 137.5 kDa protein in MPL1-PPC1 intergenic region.	1.2.1.3	174.57
P38992	SUR2 protein (Syringomycin response protein 2).	1.3.3.-	305.5
P36168	Hypothetical 137.5 kDa protein in MPL1-PPC1 intergenic region.	1.5.1.12	174.57
P40215	Hypothetical 62.8 kDa protein in RPS16A-TIF34 intergenic region.	1.8.1.9	111.09
P52923	Hypothetical 41.3 kDa protein in HXT17-COS10 intergenic region.	1.8.1.9	111.09
P14908	Mitochondrial replication protein MTF1 (Mitochondrial transcription factor mtTFB) (RF1023) (Mitochondrial specificity factor).	2.1.1.-	203.67
P87250	Mitochondrial replication protein MTF1 (Mitochondrial transcription factor MTTFB).	2.1.1.-	203.67
