# Supplementary material for: Association algorithm to mine the rules that govern enzyme definition and to classify protein sequences
Source: BMC Bioinformatics. 2006 Jun 15;7:304. doi: 10.1186/1471-2105-7-304 (PMC1552092; doi:10.1186/1471-2105-7-304)
Supplement: Additional File 1 — All rules generated from the fungus training dataset. [file 1471-2105-7-304-S1.doc]

| sn | iprs | support1 (iprs) | ec_id | support2 (ec_id) | confidence | lift |
| --- | --- | --- | --- | --- | --- | --- |
| 1 | IPR000873,IPR001031,IPR001242,IPR006163 | 4 | 6.3.2.26 | 4 | 1 | 916.5 |
| 2 | IPR001031,IPR001242,IPR006163 | 4 | 6.3.2.26 | 4 | 1 | 916.5 |
| 3 | IPR000873,IPR001031,IPR006163 | 4 | 6.3.2.26 | 4 | 1 | 916.5 |
| 4 | IPR000873,IPR001031,IPR001242 | 4 | 6.3.2.26 | 4 | 1 | 916.5 |
| 5 | IPR002314,IPR002317 | 4 | 6.1.1.11 | 4 | 1 | 916.5 |
| 6 | IPR001926,IPR002028 | 4 | 4.2.1.20 | 4 | 1 | 916.5 |
| 7 | IPR001031,IPR001242 | 4 | 6.3.2.26 | 4 | 1 | 916.5 |
| 8 | IPR000873,IPR001031 | 4 | 6.3.2.26 | 4 | 1 | 916.5 |
| 9 | IPR000850,IPR007862 | 4 | 2.7.4.3 | 4 | 1 | 916.5 |
| 10 | IPR007862 | 4 | 2.7.4.3 | 4 | 1 | 916.5 |
| 11 | IPR004308 | 4 | 6.3.2.2 | 4 | 1 | 916.5 |
| 12 | IPR003171 | 4 | 1.5.1.20 | 4 | 1 | 916.5 |
| 13 | IPR002934 | 4 | 2.7.7.19 | 4 | 1 | 916.5 |
| 14 | IPR002317 | 4 | 6.1.1.11 | 4 | 1 | 916.5 |
| 15 | IPR002042 | 4 | 1.7.3.3 | 4 | 1 | 916.5 |
| 16 | IPR002028 | 4 | 4.2.1.20 | 4 | 1 | 916.5 |
| 17 | IPR001731 | 4 | 4.2.1.24 | 4 | 1 | 916.5 |
| 18 | IPR001604 | 4 | 3.1.30.- | 4 | 1 | 916.5 |
| 19 | IPR001585 | 4 | 2.2.1.2 | 4 | 1 | 916.5 |
| 20 | IPR001544 | 4 | 2.6.1.42 | 4 | 1 | 916.5 |
| 21 | IPR001474 | 4 | 3.5.4.16 | 4 | 1 | 916.5 |
| 22 | IPR001382 | 4 | 3.2.1.113 | 4 | 1 | 916.5 |
| 23 | IPR000977 | 4 | 6.5.1.1 | 4 | 1 | 916.5 |
| 24 | IPR000304 | 4 | 1.5.1.2 | 4 | 1 | 916.5 |
| 25 | IPR005843,IPR005844,IPR005845,IPR005846 | 5 | 5.4.2.2 | 5 | 1 | 733.2 |
| 26 | IPR005844,IPR005845,IPR005846 | 5 | 5.4.2.2 | 5 | 1 | 733.2 |
| 27 | IPR005843,IPR005845,IPR005846 | 5 | 5.4.2.2 | 5 | 1 | 733.2 |
| 28 | IPR005843,IPR005844,IPR005846 | 5 | 5.4.2.2 | 5 | 1 | 733.2 |
| 29 | IPR006114,IPR006115 | 5 | 1.1.1.44 | 5 | 1 | 733.2 |
| 30 | IPR005845,IPR005846 | 5 | 5.4.2.2 | 5 | 1 | 733.2 |
| 31 | IPR005844,IPR005846 | 5 | 5.4.2.2 | 5 | 1 | 733.2 |
| 32 | IPR005843,IPR005846 | 5 | 5.4.2.2 | 5 | 1 | 733.2 |
| 33 | IPR009447 | 5 | 2.3.-.- | 5 | 1 | 733.2 |
| 34 | IPR006115 | 5 | 1.1.1.44 | 5 | 1 | 733.2 |
| 35 | IPR006114 | 5 | 1.1.1.44 | 5 | 1 | 733.2 |
| 36 | IPR005846 | 5 | 5.4.2.2 | 5 | 1 | 733.2 |
| 37 | IPR002133 | 5 | 2.5.1.6 | 5 | 1 | 733.2 |
| 38 | IPR001568 | 5 | 3.1.27.1 | 5 | 1 | 733.2 |
| 39 | IPR001564 | 5 | 2.7.4.6 | 5 | 1 | 733.2 |
| 40 | IPR001106 | 5 | 4.3.1.5 | 5 | 1 | 733.2 |
| 41 | IPR000183 | 5 | 4.1.1.17 | 5 | 1 | 733.2 |
| 42 | IPR000056 | 5 | 5.1.3.1 | 5 | 1 | 733.2 |
| 43 | IPR004113,IPR006094 | 5 | 1.1.2.4 | 4 | 0.8 | 733.2 |
| 44 | IPR001031,IPR006163 | 5 | 6.3.2.26 | 4 | 0.8 | 733.2 |
| 45 | IPR004113 | 5 | 1.1.2.4 | 4 | 0.8 | 733.2 |
| 46 | IPR002227 | 5 | 1.14.18.1 | 4 | 0.8 | 733.2 |
| 47 | IPR001597 | 5 | 4.1.2.5 | 4 | 0.8 | 733.2 |
| 48 | IPR001031 | 5 | 6.3.2.26 | 4 | 0.8 | 733.2 |
| 49 | IPR000159,IPR001054,IPR001611,IPR001932 | 6 | 4.6.1.1 | 6 | 1 | 611 |
| 50 | IPR001433,IPR003097,IPR008254 | 6 | 1.6.2.4 | 6 | 1 | 611 |
| 51 | IPR001054,IPR001611,IPR001932 | 6 | 4.6.1.1 | 6 | 1 | 611 |
| 52 | IPR000159,IPR001611,IPR001932 | 6 | 4.6.1.1 | 6 | 1 | 611 |
| 53 | IPR000159,IPR001054,IPR001932 | 6 | 4.6.1.1 | 6 | 1 | 611 |
| 54 | IPR000159,IPR001054,IPR001611 | 6 | 4.6.1.1 | 6 | 1 | 611 |
| 55 | IPR005849,IPR005850 | 6 | 2.7.7.12 | 6 | 1 | 611 |
| 56 | IPR004006,IPR004007 | 6 | 2.7.1.29 | 6 | 1 | 611 |
| 57 | IPR003097,IPR008254 | 6 | 1.6.2.4 | 6 | 1 | 611 |
| 58 | IPR001611,IPR001932 | 6 | 4.6.1.1 | 6 | 1 | 611 |
| 59 | IPR001433,IPR008254 | 6 | 1.6.2.4 | 6 | 1 | 611 |
| 60 | IPR001054,IPR001932 | 6 | 4.6.1.1 | 6 | 1 | 611 |
| 61 | IPR001054,IPR001611 | 6 | 4.6.1.1 | 6 | 1 | 611 |
| 62 | IPR000159,IPR001932 | 6 | 4.6.1.1 | 6 | 1 | 611 |
| 63 | IPR000159,IPR001611 | 6 | 4.6.1.1 | 6 | 1 | 611 |
| 64 | IPR000159,IPR001054 | 6 | 4.6.1.1 | 6 | 1 | 611 |
| 65 | IPR010458 | 6 | 4.2.3.6 | 6 | 1 | 611 |
| 66 | IPR008254 | 6 | 1.6.2.4 | 6 | 1 | 611 |
| 67 | IPR007266 | 6 | 1.8.4.- | 6 | 1 | 611 |
| 68 | IPR006034 | 6 | 3.5.1.1 | 6 | 1 | 611 |
| 69 | IPR005850 | 6 | 2.7.7.12 | 6 | 1 | 611 |
| 70 | IPR005849 | 6 | 2.7.7.12 | 6 | 1 | 611 |
| 71 | IPR004299 | 6 | 2.3.1.26 | 6 | 1 | 611 |
| 72 | IPR004007 | 6 | 2.7.1.29 | 6 | 1 | 611 |
| 73 | IPR004006 | 6 | 2.7.1.29 | 6 | 1 | 611 |
| 74 | IPR002650 | 6 | 2.7.7.4 | 6 | 1 | 611 |
| 75 | IPR002205 | 6 | 5.99.1.3 | 6 | 1 | 611 |
| 76 | IPR001796 | 6 | 1.5.1.3 | 6 | 1 | 611 |
| 77 | IPR001465 | 6 | 2.3.3.9 | 6 | 1 | 611 |
| 78 | IPR001282 | 6 | 1.1.1.49 | 6 | 1 | 611 |
| 79 | IPR001054 | 6 | 4.6.1.1 | 6 | 1 | 611 |
| 80 | IPR000159 | 6 | 4.6.1.1 | 6 | 1 | 611 |
| 81 | IPR001241,IPR002205,IPR003594 | 5 | 5.99.1.3 | 5 | 1 | 611 |
| 82 | IPR002205,IPR003594 | 5 | 5.99.1.3 | 5 | 1 | 611 |
| 83 | IPR001241,IPR003594 | 5 | 5.99.1.3 | 5 | 1 | 611 |
| 84 | IPR001241,IPR002205 | 5 | 5.99.1.3 | 5 | 1 | 611 |
| 85 | IPR006218 | 5 | 2.5.1.54 | 5 | 1 | 611 |
| 86 | IPR001241 | 5 | 5.99.1.3 | 5 | 1 | 611 |
| 87 | IPR000407 | 6 | 3.6.1.42 | 5 | 0.83 | 611 |
| 88 | IPR000873,IPR001242,IPR006163 | 6 | 6.3.2.26 | 4 | 0.67 | 611 |
| 89 | IPR003135,IPR005811 | 4 | 6.2.1.4 | 4 | 1 | 611 |
| 90 | IPR002650,IPR002891 | 4 | 2.7.7.4 | 4 | 1 | 611 |
| 91 | IPR001631,IPR008336 | 4 | 5.99.1.2 | 4 | 1 | 611 |
| 92 | IPR001242,IPR006163 | 6 | 6.3.2.26 | 4 | 0.67 | 611 |
| 93 | IPR000873,IPR001242 | 6 | 6.3.2.26 | 4 | 0.67 | 611 |
| 94 | IPR008336 | 4 | 5.99.1.2 | 4 | 1 | 611 |
| 95 | IPR001631 | 4 | 5.99.1.2 | 4 | 1 | 611 |
| 96 | IPR001345 | 6 | 5.4.2.1 | 4 | 0.67 | 611 |
| 97 | IPR001242 | 6 | 6.3.2.26 | 4 | 0.67 | 611 |
| 98 | IPR000850 | 6 | 2.7.4.3 | 4 | 0.67 | 611 |
| 99 | IPR000511 | 6 | 4.4.1.17 | 4 | 0.67 | 611 |
| 100 | IPR000089,IPR000891,IPR003379,IPR005479,IPR005481,IPR005482 | 7 | 6.4.1.1 | 7 | 1 | 523.71 |
| 101 | IPR000891,IPR003379,IPR005479,IPR005481,IPR005482 | 7 | 6.4.1.1 | 7 | 1 | 523.71 |
| 102 | IPR000089,IPR003379,IPR005479,IPR005481,IPR005482 | 7 | 6.4.1.1 | 7 | 1 | 523.71 |
| 103 | IPR000089,IPR000891,IPR005479,IPR005481,IPR005482 | 7 | 6.4.1.1 | 7 | 1 | 523.71 |
| 104 | IPR000089,IPR000891,IPR003379,IPR005481,IPR005482 | 7 | 6.4.1.1 | 7 | 1 | 523.71 |
| 105 | IPR000089,IPR000891,IPR003379,IPR005479,IPR005482 | 7 | 6.4.1.1 | 7 | 1 | 523.71 |
| 106 | IPR000089,IPR000891,IPR003379,IPR005479,IPR005481 | 7 | 6.4.1.1 | 7 | 1 | 523.71 |
| 107 | IPR003379,IPR005479,IPR005481,IPR005482 | 7 | 6.4.1.1 | 7 | 1 | 523.71 |
| 108 | IPR000891,IPR005479,IPR005481,IPR005482 | 7 | 6.4.1.1 | 7 | 1 | 523.71 |
| 109 | IPR000891,IPR003379,IPR005481,IPR005482 | 7 | 6.4.1.1 | 7 | 1 | 523.71 |
| 110 | IPR000891,IPR003379,IPR005479,IPR005482 | 7 | 6.4.1.1 | 7 | 1 | 523.71 |
| 111 | IPR000891,IPR003379,IPR005479,IPR005481 | 7 | 6.4.1.1 | 7 | 1 | 523.71 |
| 112 | IPR000089,IPR005479,IPR005481,IPR005482 | 7 | 6.4.1.1 | 7 | 1 | 523.71 |
| 113 | IPR000089,IPR003379,IPR005481,IPR005482 | 7 | 6.4.1.1 | 7 | 1 | 523.71 |
| 114 | IPR000089,IPR003379,IPR005479,IPR005482 | 7 | 6.4.1.1 | 7 | 1 | 523.71 |
| 115 | IPR000089,IPR003379,IPR005479,IPR005481 | 7 | 6.4.1.1 | 7 | 1 | 523.71 |
| 116 | IPR000089,IPR000891,IPR005481,IPR005482 | 7 | 6.4.1.1 | 7 | 1 | 523.71 |
| 117 | IPR000089,IPR000891,IPR005479,IPR005482 | 7 | 6.4.1.1 | 7 | 1 | 523.71 |
| 118 | IPR000089,IPR000891,IPR005479,IPR005481 | 7 | 6.4.1.1 | 7 | 1 | 523.71 |
| 119 | IPR000089,IPR000891,IPR003379,IPR005482 | 7 | 6.4.1.1 | 7 | 1 | 523.71 |
| 120 | IPR000089,IPR000891,IPR003379,IPR005481 | 7 | 6.4.1.1 | 7 | 1 | 523.71 |
| 121 | IPR000089,IPR000891,IPR003379,IPR005479 | 7 | 6.4.1.1 | 7 | 1 | 523.71 |
| 122 | IPR005479,IPR005481,IPR005482 | 7 | 6.4.1.1 | 7 | 1 | 523.71 |
| 123 | IPR003379,IPR005481,IPR005482 | 7 | 6.4.1.1 | 7 | 1 | 523.71 |
| 124 | IPR003379,IPR005479,IPR005482 | 7 | 6.4.1.1 | 7 | 1 | 523.71 |
| 125 | IPR003379,IPR005479,IPR005481 | 7 | 6.4.1.1 | 7 | 1 | 523.71 |
| 126 | IPR000891,IPR005481,IPR005482 | 7 | 6.4.1.1 | 7 | 1 | 523.71 |
| 127 | IPR000891,IPR005479,IPR005482 | 7 | 6.4.1.1 | 7 | 1 | 523.71 |
| 128 | IPR000891,IPR005479,IPR005481 | 7 | 6.4.1.1 | 7 | 1 | 523.71 |
| 129 | IPR000891,IPR003379,IPR005482 | 7 | 6.4.1.1 | 7 | 1 | 523.71 |
| 130 | IPR000891,IPR003379,IPR005481 | 7 | 6.4.1.1 | 7 | 1 | 523.71 |
| 131 | IPR000891,IPR003379,IPR005479 | 7 | 6.4.1.1 | 7 | 1 | 523.71 |
| 132 | IPR000089,IPR005481,IPR005482 | 7 | 6.4.1.1 | 7 | 1 | 523.71 |
| 133 | IPR000089,IPR005479,IPR005482 | 7 | 6.4.1.1 | 7 | 1 | 523.71 |
| 134 | IPR000089,IPR005479,IPR005481 | 7 | 6.4.1.1 | 7 | 1 | 523.71 |
| 135 | IPR000089,IPR003379,IPR005482 | 7 | 6.4.1.1 | 7 | 1 | 523.71 |
| 136 | IPR000089,IPR003379,IPR005481 | 7 | 6.4.1.1 | 7 | 1 | 523.71 |
| 137 | IPR000089,IPR003379,IPR005479 | 7 | 6.4.1.1 | 7 | 1 | 523.71 |
| 138 | IPR000089,IPR000891,IPR005482 | 7 | 6.4.1.1 | 7 | 1 | 523.71 |
| 139 | IPR000089,IPR000891,IPR005481 | 7 | 6.4.1.1 | 7 | 1 | 523.71 |
| 140 | IPR000089,IPR000891,IPR005479 | 7 | 6.4.1.1 | 7 | 1 | 523.71 |
| 141 | IPR000089,IPR000891,IPR003379 | 7 | 6.4.1.1 | 7 | 1 | 523.71 |
| 142 | IPR006131,IPR006132 | 7 | 2.1.3.3 | 7 | 1 | 523.71 |
| 143 | IPR005481,IPR005482 | 7 | 6.4.1.1 | 7 | 1 | 523.71 |
| 144 | IPR005479,IPR005482 | 7 | 6.4.1.1 | 7 | 1 | 523.71 |
| 145 | IPR003379,IPR005482 | 7 | 6.4.1.1 | 7 | 1 | 523.71 |
| 146 | IPR003379,IPR005481 | 7 | 6.4.1.1 | 7 | 1 | 523.71 |
| 147 | IPR003379,IPR005479 | 7 | 6.4.1.1 | 7 | 1 | 523.71 |
| 148 | IPR000891,IPR005482 | 7 | 6.4.1.1 | 7 | 1 | 523.71 |
| 149 | IPR000891,IPR005481 | 7 | 6.4.1.1 | 7 | 1 | 523.71 |
| 150 | IPR000891,IPR005479 | 7 | 6.4.1.1 | 7 | 1 | 523.71 |
| 151 | IPR000891,IPR003379 | 7 | 6.4.1.1 | 7 | 1 | 523.71 |
| 152 | IPR000644,IPR001093 | 7 | 1.1.1.205 | 7 | 1 | 523.71 |
| 153 | IPR000089,IPR005482 | 7 | 6.4.1.1 | 7 | 1 | 523.71 |
| 154 | IPR000089,IPR005481 | 7 | 6.4.1.1 | 7 | 1 | 523.71 |
| 155 | IPR000089,IPR005479 | 7 | 6.4.1.1 | 7 | 1 | 523.71 |
| 156 | IPR000089,IPR003379 | 7 | 6.4.1.1 | 7 | 1 | 523.71 |
| 157 | IPR000089,IPR000891 | 7 | 6.4.1.1 | 7 | 1 | 523.71 |
| 158 | IPR000031,IPR003135 | 7 | 4.1.1.21 | 7 | 1 | 523.71 |
| 159 | IPR006677 | 7 | 3.1.27.9 | 7 | 1 | 523.71 |
| 160 | IPR006132 | 7 | 2.1.3.3 | 7 | 1 | 523.71 |
| 161 | IPR006131 | 7 | 2.1.3.3 | 7 | 1 | 523.71 |
| 162 | IPR005482 | 7 | 6.4.1.1 | 7 | 1 | 523.71 |
| 163 | IPR005198 | 7 | 3.2.1.101 | 7 | 1 | 523.71 |
| 164 | IPR003702 | 7 | 3.1.2.1 | 7 | 1 | 523.71 |
| 165 | IPR003440 | 7 | 2.4.1.34 | 7 | 1 | 523.71 |
| 166 | IPR003379 | 7 | 6.4.1.1 | 7 | 1 | 523.71 |
| 167 | IPR002734 | 7 | 1.1.1.193 | 7 | 1 | 523.71 |
| 168 | IPR002202 | 7 | 1.1.1.34 | 7 | 1 | 523.71 |
| 169 | IPR001636 | 7 | 6.3.2.6 | 7 | 1 | 523.71 |
| 170 | IPR001272 | 7 | 4.1.1.49 | 7 | 1 | 523.71 |
| 171 | IPR001155 | 7 | 1.6.99.1 | 7 | 1 | 523.71 |
| 172 | IPR001093 | 7 | 1.1.1.205 | 7 | 1 | 523.71 |
| 173 | IPR000771 | 7 | 4.1.2.13 | 7 | 1 | 523.71 |
| 174 | IPR000398 | 7 | 2.1.1.45 | 7 | 1 | 523.71 |
| 175 | IPR000031 | 7 | 4.1.1.21 | 7 | 1 | 523.71 |
| 176 | IPR004045,IPR004046 | 7 | 2.5.1.18 | 6 | 0.86 | 523.71 |
| 177 | IPR004046 | 7 | 2.5.1.18 | 6 | 0.86 | 523.71 |
| 178 | IPR004045 | 7 | 2.5.1.18 | 6 | 0.86 | 523.71 |
| 179 | IPR002772 | 6 | 3.2.1.21 | 6 | 1 | 523.71 |
| 180 | IPR001764 | 6 | 3.2.1.21 | 6 | 1 | 523.71 |
| 181 | IPR001611 | 7 | 4.6.1.1 | 6 | 0.86 | 523.71 |
| 182 | IPR001764,IPR002772 | 5 | 3.2.1.21 | 5 | 1 | 523.71 |
| 183 | IPR002022 | 7 | 4.2.2.10 | 5 | 0.71 | 523.71 |
| 184 | IPR000357,IPR000403,IPR003151,IPR003152 | 4 | 2.7.1.137 | 4 | 1 | 523.71 |
| 185 | IPR005479,IPR005480,IPR005481 | 4 | 6.3.5.5 | 4 | 1 | 523.71 |
| 186 | IPR000403,IPR003151,IPR003152 | 4 | 2.7.1.137 | 4 | 1 | 523.71 |
| 187 | IPR000357,IPR003151,IPR003152 | 4 | 2.7.1.137 | 4 | 1 | 523.71 |
| 188 | IPR000357,IPR000403,IPR003152 | 4 | 2.7.1.137 | 4 | 1 | 523.71 |
| 189 | IPR000357,IPR000403,IPR003151 | 4 | 2.7.1.137 | 4 | 1 | 523.71 |
| 190 | IPR005480,IPR005481 | 4 | 6.3.5.5 | 4 | 1 | 523.71 |
| 191 | IPR005479,IPR005480 | 4 | 6.3.5.5 | 4 | 1 | 523.71 |
| 192 | IPR003151,IPR003152 | 4 | 2.7.1.137 | 4 | 1 | 523.71 |
| 193 | IPR000403,IPR003152 | 4 | 2.7.1.137 | 4 | 1 | 523.71 |
| 194 | IPR000403,IPR003151 | 4 | 2.7.1.137 | 4 | 1 | 523.71 |
| 195 | IPR000357,IPR003152 | 4 | 2.7.1.137 | 4 | 1 | 523.71 |
| 196 | IPR000357,IPR003151 | 4 | 2.7.1.137 | 4 | 1 | 523.71 |
| 197 | IPR000357,IPR000403 | 4 | 2.7.1.137 | 4 | 1 | 523.71 |
| 198 | IPR005480 | 4 | 6.3.5.5 | 4 | 1 | 523.71 |
| 199 | IPR003152 | 4 | 2.7.1.137 | 4 | 1 | 523.71 |
| 200 | IPR003151 | 4 | 2.7.1.137 | 4 | 1 | 523.71 |
| 203 | IPR003594 | 6 | 5.99.1.3 | 5 | 0.83 | 509.17 |
| 204 | IPR000572,IPR001199,IPR001433,IPR005066,IPR008333 | 8 | 1.7.1.3 | 8 | 1 | 458.25 |
| 205 | IPR001199,IPR001433,IPR005066,IPR008333 | 8 | 1.7.1.3 | 8 | 1 | 458.25 |
| 206 | IPR000572,IPR001433,IPR005066,IPR008333 | 8 | 1.7.1.3 | 8 | 1 | 458.25 |
| 207 | IPR000572,IPR001199,IPR005066,IPR008333 | 8 | 1.7.1.3 | 8 | 1 | 458.25 |
| 208 | IPR000572,IPR001199,IPR001433,IPR008333 | 8 | 1.7.1.3 | 8 | 1 | 458.25 |
| 209 | IPR000572,IPR001199,IPR001433,IPR005066 | 8 | 1.7.1.3 | 8 | 1 | 458.25 |
| 210 | IPR001433,IPR005066,IPR008333 | 8 | 1.7.1.3 | 8 | 1 | 458.25 |
| 211 | IPR001199,IPR005066,IPR008333 | 8 | 1.7.1.3 | 8 | 1 | 458.25 |
| 212 | IPR001199,IPR001433,IPR008333 | 8 | 1.7.1.3 | 8 | 1 | 458.25 |
| 213 | IPR001199,IPR001433,IPR005066 | 8 | 1.7.1.3 | 8 | 1 | 458.25 |
| 214 | IPR000572,IPR005066,IPR008333 | 8 | 1.7.1.3 | 8 | 1 | 458.25 |
| 215 | IPR000572,IPR001433,IPR008333 | 8 | 1.7.1.3 | 8 | 1 | 458.25 |
| 216 | IPR000572,IPR001433,IPR005066 | 8 | 1.7.1.3 | 8 | 1 | 458.25 |
| 217 | IPR000572,IPR001199,IPR008333 | 8 | 1.7.1.3 | 8 | 1 | 458.25 |
| 218 | IPR000572,IPR001199,IPR005066 | 8 | 1.7.1.3 | 8 | 1 | 458.25 |
| 219 | IPR000572,IPR001199,IPR001433 | 8 | 1.7.1.3 | 8 | 1 | 458.25 |
| 220 | IPR007698,IPR007886 | 8 | 1.5.1.7 | 8 | 1 | 458.25 |
| 221 | IPR006109,IPR011128 | 8 | 1.1.1.8 | 8 | 1 | 458.25 |
| 222 | IPR005066,IPR008333 | 8 | 1.7.1.3 | 8 | 1 | 458.25 |
| 223 | IPR001433,IPR005066 | 8 | 1.7.1.3 | 8 | 1 | 458.25 |
| 224 | IPR001199,IPR008333 | 8 | 1.7.1.3 | 8 | 1 | 458.25 |
| 225 | IPR001199,IPR005066 | 8 | 1.7.1.3 | 8 | 1 | 458.25 |
| 226 | IPR001199,IPR001433 | 8 | 1.7.1.3 | 8 | 1 | 458.25 |
| 227 | IPR000991,IPR004468 | 8 | 6.3.4.2 | 8 | 1 | 458.25 |
| 228 | IPR000572,IPR008333 | 8 | 1.7.1.3 | 8 | 1 | 458.25 |
| 229 | IPR000572,IPR005066 | 8 | 1.7.1.3 | 8 | 1 | 458.25 |
| 230 | IPR000572,IPR001433 | 8 | 1.7.1.3 | 8 | 1 | 458.25 |
| 231 | IPR000572,IPR001199 | 8 | 1.7.1.3 | 8 | 1 | 458.25 |
| 232 | IPR011128 | 8 | 1.1.1.8 | 8 | 1 | 458.25 |
| 233 | IPR007886 | 8 | 1.5.1.7 | 8 | 1 | 458.25 |
| 234 | IPR007698 | 8 | 1.5.1.7 | 8 | 1 | 458.25 |
| 235 | IPR006109 | 8 | 1.1.1.8 | 8 | 1 | 458.25 |
| 236 | IPR005066 | 8 | 1.7.1.3 | 8 | 1 | 458.25 |
| 237 | IPR004468 | 8 | 6.3.4.2 | 8 | 1 | 458.25 |
| 238 | IPR001348 | 8 | 2.4.2.17 | 8 | 1 | 458.25 |
| 239 | IPR001250 | 8 | 5.3.1.8 | 8 | 1 | 458.25 |
| 240 | IPR000903 | 8 | 2.3.1.97 | 8 | 1 | 458.25 |
| 241 | IPR000572 | 8 | 1.7.1.3 | 8 | 1 | 458.25 |
| 242 | IPR000644 | 8 | 1.1.1.205 | 7 | 0.88 | 458.25 |
| 243 | IPR005474,IPR005475,IPR005476 | 8 | 2.2.1.1 | 6 | 0.75 | 458.25 |
| 244 | IPR005474,IPR005476 | 8 | 2.2.1.1 | 6 | 0.75 | 458.25 |
| 245 | IPR005474,IPR005475 | 8 | 2.2.1.1 | 6 | 0.75 | 458.25 |
| 246 | IPR001433,IPR003097 | 8 | 1.6.2.4 | 6 | 0.75 | 458.25 |
| 247 | IPR005474 | 8 | 2.2.1.1 | 6 | 0.75 | 458.25 |
| 248 | IPR003653 | 6 | 3.4.22.- | 6 | 1 | 458.25 |
| 249 | IPR003097 | 8 | 1.6.2.4 | 6 | 0.75 | 458.25 |
| 250 | IPR001171 | 8 | 1.3.1.70 | 6 | 0.75 | 458.25 |
| 251 | IPR004014,IPR005834,IPR006068,IPR008250 | 8 | 3.6.3.8 | 5 | 0.63 | 458.25 |
| 252 | IPR005834,IPR006068,IPR008250 | 8 | 3.6.3.8 | 5 | 0.63 | 458.25 |
| 253 | IPR004014,IPR006068,IPR008250 | 8 | 3.6.3.8 | 5 | 0.63 | 458.25 |
| 254 | IPR004014,IPR005834,IPR006068 | 8 | 3.6.3.8 | 5 | 0.63 | 458.25 |
| 255 | IPR006068,IPR008250 | 8 | 3.6.3.8 | 5 | 0.63 | 458.25 |
| 256 | IPR005834,IPR006068 | 8 | 3.6.3.8 | 5 | 0.63 | 458.25 |
| 257 | IPR004014,IPR006068 | 8 | 3.6.3.8 | 5 | 0.63 | 458.25 |
| 258 | IPR006068 | 8 | 3.6.3.8 | 5 | 0.63 | 458.25 |
| 259 | IPR000192 | 8 | 2.8.1.7 | 5 | 0.63 | 458.25 |
| 260 | IPR000073 | 8 | 2.3.1.31 | 5 | 0.63 | 458.25 |
| 261 | IPR006094,IPR007173 | 9 | 1.1.3.37 | 9 | 1 | 407.33 |
| 262 | IPR007173 | 9 | 1.1.3.37 | 9 | 1 | 407.33 |
| 263 | IPR006062 | 9 | 5.3.1.16 | 9 | 1 | 407.33 |
| 264 | IPR002916 | 9 | 1.16.1.7 | 9 | 1 | 407.33 |
| 265 | IPR002773 | 9 | 2.5.1.46 | 9 | 1 | 407.33 |
| 266 | IPR001672 | 9 | 5.3.1.9 | 9 | 1 | 407.33 |
| 267 | IPR001375 | 9 | 3.4.14.- | 9 | 1 | 407.33 |
| 268 | IPR001339 | 9 | 2.7.7.50 | 9 | 1 | 407.33 |
| 269 | IPR000860 | 9 | 2.5.1.61 | 9 | 1 | 407.33 |
| 270 | IPR002020 | 9 | 2.3.3.1 | 8 | 0.89 | 407.33 |
| 271 | IPR006035 | 9 | 3.5.3.1 | 7 | 0.78 | 407.33 |
| 272 | IPR001312 | 9 | 2.7.1.1 | 7 | 0.78 | 407.33 |
| 273 | IPR000873,IPR006163,IPR010080 | 6 | 1.2.1.31 | 6 | 1 | 407.33 |
| 274 | IPR006163,IPR010080 | 6 | 1.2.1.31 | 6 | 1 | 407.33 |
| 275 | IPR000873,IPR010080 | 6 | 1.2.1.31 | 6 | 1 | 407.33 |
| 276 | IPR010080 | 6 | 1.2.1.31 | 6 | 1 | 407.33 |
| 277 | IPR005811 | 9 | 6.2.1.4 | 6 | 0.67 | 407.33 |
| 293 | IPR001375,IPR002469 | 4 | 3.4.14.- | 4 | 1 | 407.33 |
| 297 | IPR002469 | 4 | 3.4.14.- | 4 | 1 | 407.33 |
| 301 | IPR001930 | 8 | 3.4.11.- | 6 | 0.75 | 392.79 |
| 302 | IPR003608 | 10 | 2.4.1.109 | 10 | 1 | 366.6 |
| 303 | IPR001240 | 10 | 5.3.1.24 | 10 | 1 | 366.6 |
| 304 | IPR003342,IPR003608 | 9 | 2.4.1.109 | 9 | 1 | 366.6 |
| 305 | IPR003342 | 9 | 2.4.1.109 | 9 | 1 | 366.6 |
| 306 | IPR002060 | 10 | 2.5.1.21 | 9 | 0.9 | 366.6 |
| 307 | IPR001327,IPR004099 | 10 | 1.8.1.7 | 8 | 0.8 | 366.6 |
| 308 | IPR004099 | 10 | 1.8.1.7 | 8 | 0.8 | 366.6 |
| 309 | IPR001041,IPR001450 | 7 | 1.3.5.1 | 7 | 1 | 366.6 |
| 310 | IPR001450 | 7 | 1.3.5.1 | 7 | 1 | 366.6 |
| 311 | IPR000403 | 10 | 2.7.1.137 | 7 | 0.7 | 366.6 |
| 312 | IPR005475,IPR005476 | 10 | 2.2.1.1 | 6 | 0.6 | 366.6 |
| 313 | IPR005476 | 10 | 2.2.1.1 | 6 | 0.6 | 366.6 |
| 314 | IPR002891 | 10 | 2.7.1.25 | 6 | 0.6 | 366.6 |
| 315 | IPR000788 | 5 | 1.17.4.1 | 5 | 1 | 366.6 |
| 317 | IPR000358 | 5 | 1.17.4.1 | 5 | 1 | 366.6 |
| 319 | IPR000788,IPR005144 | 4 | 1.17.4.1 | 4 | 1 | 366.6 |
| 320 | IPR005144 | 4 | 1.17.4.1 | 4 | 1 | 366.6 |
| 325 | IPR000357 | 6 | 2.7.1.137 | 4 | 0.67 | 349.14 |
| 326 | IPR008180 | 11 | 3.6.1.23 | 11 | 1 | 333.27 |
| 327 | IPR003408,IPR004839 | 10 | 2.3.1.37 | 10 | 1 | 333.27 |
| 328 | IPR003408 | 10 | 2.3.1.37 | 10 | 1 | 333.27 |
| 329 | IPR001661,IPR011120 | 9 | 3.2.1.28 | 9 | 1 | 333.27 |
| 330 | IPR011120 | 9 | 3.2.1.28 | 9 | 1 | 333.27 |
| 331 | IPR001661 | 9 | 3.2.1.28 | 9 | 1 | 333.27 |
| 332 | IPR001433,IPR008333 | 11 | 1.7.1.3 | 8 | 0.73 | 333.27 |
| 333 | IPR008333 | 11 | 1.7.1.3 | 8 | 0.73 | 333.27 |
| 334 | IPR005479,IPR005481 | 11 | 6.4.1.1 | 7 | 0.64 | 333.27 |
| 335 | IPR005481 | 11 | 6.4.1.1 | 7 | 0.64 | 333.27 |
| 336 | IPR005479 | 11 | 6.4.1.1 | 7 | 0.64 | 333.27 |
| 337 | IPR003135 | 11 | 4.1.1.21 | 7 | 0.64 | 333.27 |
| 341 | IPR008162 | 12 | 3.6.1.1 | 12 | 1 | 305.5 |
| 342 | IPR002685 | 12 | 2.4.1.131 | 12 | 1 | 305.5 |
| 343 | IPR000023 | 12 | 2.7.1.11 | 12 | 1 | 305.5 |
| 344 | IPR000569 | 10 | 6.3.2.- | 10 | 1 | 305.5 |
| 345 | IPR006088 | 12 | 1.3.3.- | 9 | 0.75 | 305.5 |
| 346 | IPR000182,IPR001487 | 7 | 2.3.1.48 | 7 | 1 | 305.5 |
| 347 | IPR001487 | 7 | 2.3.1.48 | 7 | 1 | 305.5 |
| 349 | IPR000569,IPR010309,IPR010314 | 4 | 6.3.2.- | 4 | 1 | 305.5 |
| 350 | IPR000008,IPR000569,IPR001202 | 4 | 6.3.2.- | 4 | 1 | 305.5 |
| 351 | IPR010309,IPR010314 | 4 | 6.3.2.- | 4 | 1 | 305.5 |
| 353 | IPR000569,IPR010314 | 4 | 6.3.2.- | 4 | 1 | 305.5 |
| 354 | IPR000569,IPR010309 | 4 | 6.3.2.- | 4 | 1 | 305.5 |
| 355 | IPR000569,IPR001202 | 4 | 6.3.2.- | 4 | 1 | 305.5 |
| 356 | IPR000008,IPR001202 | 4 | 6.3.2.- | 4 | 1 | 305.5 |
| 357 | IPR000008,IPR000569 | 4 | 6.3.2.- | 4 | 1 | 305.5 |
| 358 | IPR010314 | 4 | 6.3.2.- | 4 | 1 | 305.5 |
| 359 | IPR010309 | 4 | 6.3.2.- | 4 | 1 | 305.5 |
| 360 | IPR001041 | 9 | 1.3.5.1 | 7 | 0.78 | 285.13 |
| 361 | IPR001223 | 13 | 3.2.1.14 | 13 | 1 | 282 |
| 362 | IPR001085 | 13 | 2.1.2.1 | 13 | 1 | 282 |
| 363 | IPR000941 | 13 | 4.2.1.11 | 13 | 1 | 282 |
| 364 | IPR000111 | 13 | 3.2.1.22 | 13 | 1 | 282 |
| 365 | IPR006662 | 13 | 5.3.4.1 | 12 | 0.92 | 282 |
| 366 | IPR000991 | 13 | 6.3.4.2 | 8 | 0.62 | 282 |
| 369 | IPR001223,IPR005089 | 4 | 3.2.1.14 | 4 | 1 | 282 |
| 370 | IPR005089 | 4 | 3.2.1.14 | 4 | 1 | 282 |
| 372 | IPR002655 | 14 | 1.3.3.6 | 14 | 1 | 261.86 |
| 373 | IPR000026 | 14 | 3.1.27.3 | 13 | 0.93 | 261.86 |
| 374 | IPR002655,IPR006090 | 12 | 1.3.3.6 | 12 | 1 | 261.86 |
| 375 | IPR006090 | 12 | 1.3.3.6 | 12 | 1 | 261.86 |
| 376 | IPR001362 | 14 | 3.2.1.26 | 12 | 0.86 | 261.86 |
| 377 | IPR003000 | 11 | 3.5.1.- | 11 | 1 | 261.86 |
| 378 | IPR012000,IPR012001 | 14 | 4.1.1.1 | 9 | 0.64 | 261.86 |
| 379 | IPR012001 | 14 | 4.1.1.1 | 9 | 0.64 | 261.86 |
| 380 | IPR012000 | 14 | 4.1.1.1 | 9 | 0.64 | 261.86 |
| 381 | IPR006094 | 14 | 1.1.3.37 | 9 | 0.64 | 261.86 |
| 384 | IPR003000,IPR007654 | 5 | 3.5.1.- | 5 | 1 | 261.86 |
| 385 | IPR007654 | 5 | 3.5.1.- | 5 | 1 | 261.86 |
| 391 | IPR001295 | 15 | 1.3.3.1 | 15 | 1 | 244.4 |
| 392 | IPR000652 | 15 | 5.3.1.1 | 15 | 1 | 244.4 |
| 393 | IPR000675 | 15 | 3.1.1.74 | 14 | 0.93 | 244.4 |
| 397 | IPR006096,IPR006097 | 16 | 1.4.1.4 | 16 | 1 | 229.13 |
| 398 | IPR006097 | 16 | 1.4.1.4 | 16 | 1 | 229.13 |
| 399 | IPR001697 | 16 | 2.7.1.40 | 16 | 1 | 229.13 |
| 400 | IPR002016 | 16 | 1.11.1.14 | 10 | 0.63 | 229.12 |
| 401 | IPR001247 | 10 | 3.1.13.- | 10 | 1 | 229.13 |
| 402 | IPR000340 | 7 | 3.1.3.48 | 7 | 1 | 229.13 |
| 404 | IPR000242 | 6 | 3.1.3.48 | 6 | 1 | 229.13 |
| 406 | IPR002642 | 17 | 3.1.1.5 | 17 | 1 | 215.65 |
| 407 | IPR011613 | 15 | 3.2.1.3 | 15 | 1 | 215.65 |
| 408 | IPR001830 | 17 | 2.4.1.15 | 14 | 0.82 | 215.65 |
| 412 | IPR002044,IPR011613 | 7 | 3.2.1.3 | 7 | 1 | 215.65 |
| 413 | IPR002044 | 7 | 3.2.1.3 | 7 | 1 | 215.65 |
| 417 | IPR000254,IPR001722 | 13 | 3.2.1.91 | 11 | 0.85 | 206.8 |
| 418 | IPR008146,IPR008147 | 18 | 6.3.1.2 | 18 | 1 | 203.67 |
| 419 | IPR008147 | 18 | 6.3.1.2 | 18 | 1 | 203.67 |
| 420 | IPR008146 | 18 | 6.3.1.2 | 18 | 1 | 203.67 |
| 421 | IPR000918 | 18 | 4.1.3.1 | 18 | 1 | 203.67 |
| 422 | IPR006096 | 18 | 1.4.1.4 | 16 | 0.89 | 203.67 |
| 426 | IPR001737 | 7 | 2.1.1.- | 7 | 1 | 203.67 |
| 428 | IPR002877 | 6 | 2.1.1.- | 6 | 1 | 203.67 |
| 431 | IPR001202 | 6 | 6.3.2.- | 4 | 0.67 | 203.67 |
| 432 | IPR000807 | 19 | 4.2.1.19 | 19 | 1 | 192.95 |
| 438 | IPR000334 | 4 | 3.2.1.4 | 4 | 1 | 192.95 |
| 442 | IPR011614 | 19 | 1.11.1.6 | 19 | 1 | 183.3 |
| 443 | IPR010582,IPR011614 | 9 | 1.11.1.6 | 9 | 1 | 183.3 |
| 444 | IPR010582 | 9 | 1.11.1.6 | 9 | 1 | 183.3 |
| 447 | IPR003732 | 6 | 3.1.-.- | 6 | 1 | 183.3 |
| 449 | IPR001722 | 18 | 3.2.1.91 | 13 | 0.72 | 176.51 |
| 450 | IPR001576 | 21 | 2.7.2.3 | 21 | 1 | 174.57 |
| 452 | IPR008630 | 6 | 2.4.1.- | 6 | 1 | 174.57 |
| 454 | IPR004856 | 4 | 2.4.1.- | 4 | 1 | 174.57 |
| 456 | IPR003029 | 7 | 3.1.13.- | 5 | 0.71 | 163.66 |
| 457 | IPR000743 | 23 | 3.2.1.15 | 20 | 0.87 | 159.39 |
| 461 | IPR001650 | 15 | 3.6.1.- | 15 | 1 | 152.75 |
| 462 | IPR001650,IPR011545 | 13 | 3.6.1.- | 13 | 1 | 152.75 |
| 463 | IPR011545 | 13 | 3.6.1.- | 13 | 1 | 152.75 |
| 466 | IPR001650,IPR004179,IPR011545 | 4 | 3.6.1.- | 4 | 1 | 152.75 |
| 467 | IPR004179,IPR011545 | 4 | 3.6.1.- | 4 | 1 | 152.75 |
| 468 | IPR001650,IPR004179 | 4 | 3.6.1.- | 4 | 1 | 152.75 |
| 469 | IPR004179 | 4 | 3.6.1.- | 4 | 1 | 152.75 |
| 475 | IPR005152 | 10 | 3.1.1.3 | 10 | 1 | 146.64 |
| 476 | IPR002018 | 10 | 3.1.1.3 | 10 | 1 | 146.64 |
| 478 | IPR006133,IPR006134 | 8 | 2.7.7.7 | 8 | 1 | 146.64 |
| 479 | IPR006134 | 8 | 2.7.7.7 | 8 | 1 | 146.64 |
| 480 | IPR006133 | 8 | 2.7.7.7 | 8 | 1 | 146.64 |
| 481 | IPR004868 | 8 | 2.7.7.7 | 8 | 1 | 146.64 |
| 485 | IPR001098 | 4 | 2.7.7.7 | 4 | 1 | 146.64 |
| 488 | IPR001547 | 23 | 3.2.1.58 | 18 | 0.78 | 136.62 |
| 489 | IPR001137 | 19 | 3.2.1.8 | 19 | 1 | 135.78 |
| 490 | IPR003197 | 9 | 1.10.2.2 | 9 | 1 | 135.78 |
| 491 | IPR004205 | 4 | 1.10.2.2 | 4 | 1 | 135.78 |
| 493 | IPR000608 | 28 | 6.3.2.19 | 28 | 1 | 130.93 |
| 494 | IPR000209 | 24 | 3.4.21.- | 17 | 0.71 | 129.84 |
| 495 | IPR000209,IPR010259 | 17 | 3.4.21.- | 12 | 0.71 | 129.39 |
| 496 | IPR010259 | 17 | 3.4.21.- | 12 | 0.71 | 129.39 |
| 498 | IPR002130 | 18 | 5.2.1.8 | 18 | 1 | 122.2 |
| 499 | IPR001179 | 10 | 5.2.1.8 | 10 | 1 | 122.2 |
| 501 | IPR001394,IPR001607 | 4 | 3.1.2.15 | 4 | 1 | 122.2 |
| 502 | IPR001607 | 4 | 3.1.2.15 | 4 | 1 | 122.2 |
| 503 | IPR001000 | 9 | 3.2.1.8 | 8 | 0.89 | 120.69 |
| 504 | IPR001353 | 31 | 3.4.25.1 | 31 | 1 | 118.26 |
| 507 | IPR001394 | 30 | 3.1.2.15 | 29 | 0.97 | 118.13 |
| 509 | IPR001117 | 32 | 1.10.3.2 | 25 | 0.78 | 114.56 |
| 511 | IPR001395 | 30 | 1.1.1.- | 23 | 0.77 | 112.42 |
| 514 | IPR001424 | 24 | 1.15.1.1 | 24 | 1 | 107.82 |
| 515 | IPR001189 | 10 | 1.15.1.1 | 10 | 1 | 107.82 |
| 522 | IPR001750 | 19 | 1.6.5.3 | 19 | 1 | 89.41 |
| 524 | IPR001516,IPR001750 | 9 | 1.6.5.3 | 9 | 1 | 89.41 |
| 525 | IPR001516 | 9 | 1.6.5.3 | 9 | 1 | 89.41 |
| 527 | IPR001516,IPR001750,IPR010934 | 8 | 1.6.5.3 | 8 | 1 | 89.41 |
| 528 | IPR001750,IPR010934 | 8 | 1.6.5.3 | 8 | 1 | 89.41 |
| 529 | IPR001516,IPR010934 | 8 | 1.6.5.3 | 8 | 1 | 89.41 |
| 530 | IPR010934 | 8 | 1.6.5.3 | 8 | 1 | 89.41 |
| 532 | IPR000440 | 6 | 1.6.5.3 | 6 | 1 | 89.41 |
| 533 | IPR001694 | 5 | 1.6.5.3 | 5 | 1 | 89.41 |
| 535 | IPR001457 | 5 | 1.6.5.3 | 5 | 1 | 89.41 |
| 536 | IPR001133 | 5 | 1.6.5.3 | 5 | 1 | 89.41 |
| 539 | IPR001804 | 43 | 1.1.1.85 | 29 | 0.67 | 85.26 |
| 542 | IPR004843 | 32 | 3.1.3.16 | 28 | 0.88 | 78.24 |
| 544 | IPR002680 | 17 | 1.-.-.- | 17 | 1 | 74.82 |
| 546 | IPR001269 | 4 | 1.-.-.- | 4 | 1 | 74.82 |
| 547 | IPR001754 | 50 | 4.1.1.23 | 50 | 1 | 73.32 |
| 550 | IPR004834 | 45 | 2.4.1.16 | 45 | 1 | 67.89 |
| 551 | IPR001173,IPR004834 | 13 | 2.4.1.16 | 13 | 1 | 67.89 |
| 552 | IPR004835 | 11 | 2.4.1.16 | 11 | 1 | 67.89 |
| 553 | IPR001199,IPR004835 | 8 | 2.4.1.16 | 8 | 1 | 67.89 |
| 554 | IPR001173,IPR004835 | 6 | 2.4.1.16 | 6 | 1 | 67.89 |
| 561 | IPR000173 | 59 | 1.2.1.12 | 59 | 1 | 62.14 |
| 562 | IPR000008,IPR000719,IPR000861,IPR000961,IPR002219 | 8 | 2.7.1.- | 8 | 1 | 62.14 |
| 563 | IPR000719,IPR000861,IPR000961,IPR002219 | 8 | 2.7.1.- | 8 | 1 | 62.14 |
| 564 | IPR000008,IPR000861,IPR000961,IPR002219 | 8 | 2.7.1.- | 8 | 1 | 62.14 |
| 565 | IPR000008,IPR000719,IPR000961,IPR002219 | 8 | 2.7.1.- | 8 | 1 | 62.14 |
| 566 | IPR000008,IPR000719,IPR000861,IPR002219 | 8 | 2.7.1.- | 8 | 1 | 62.14 |
| 567 | IPR000008,IPR000719,IPR000861,IPR000961 | 8 | 2.7.1.- | 8 | 1 | 62.14 |
| 568 | IPR000861,IPR000961,IPR002219 | 8 | 2.7.1.- | 8 | 1 | 62.14 |
| 569 | IPR000719,IPR000961,IPR002219 | 8 | 2.7.1.- | 8 | 1 | 62.14 |
| 570 | IPR000719,IPR000861,IPR002219 | 8 | 2.7.1.- | 8 | 1 | 62.14 |
| 571 | IPR000719,IPR000861,IPR000961 | 8 | 2.7.1.- | 8 | 1 | 62.14 |
| 572 | IPR000008,IPR000961,IPR002219 | 8 | 2.7.1.- | 8 | 1 | 62.14 |
| 573 | IPR000008,IPR000861,IPR002219 | 8 | 2.7.1.- | 8 | 1 | 62.14 |
| 574 | IPR000008,IPR000861,IPR000961 | 8 | 2.7.1.- | 8 | 1 | 62.14 |
| 575 | IPR000008,IPR000719,IPR002219 | 8 | 2.7.1.- | 8 | 1 | 62.14 |
| 576 | IPR000008,IPR000719,IPR000861 | 8 | 2.7.1.- | 8 | 1 | 62.14 |
| 577 | IPR000961,IPR002219 | 8 | 2.7.1.- | 8 | 1 | 62.14 |
| 578 | IPR000861,IPR002219 | 8 | 2.7.1.- | 8 | 1 | 62.14 |
| 579 | IPR000861,IPR000961 | 8 | 2.7.1.- | 8 | 1 | 62.14 |
| 580 | IPR000719,IPR002219 | 8 | 2.7.1.- | 8 | 1 | 62.14 |
| 581 | IPR000719,IPR000861 | 8 | 2.7.1.- | 8 | 1 | 62.14 |
| 582 | IPR000008,IPR002219 | 8 | 2.7.1.- | 8 | 1 | 62.14 |
| 583 | IPR000008,IPR000861 | 8 | 2.7.1.- | 8 | 1 | 62.14 |
| 584 | IPR002219 | 8 | 2.7.1.- | 8 | 1 | 62.14 |
| 585 | IPR000861 | 8 | 2.7.1.- | 8 | 1 | 62.14 |
| 587 | IPR001932 | 16 | 3.1.3.16 | 10 | 0.63 | 55.88 |
| 588 | IPR001173 | 21 | 2.4.1.16 | 17 | 0.81 | 54.96 |
| 589 | IPR002429,IPR011759 | 22 | 1.9.3.1 | 22 | 1 | 53.91 |
| 590 | IPR011759 | 22 | 1.9.3.1 | 22 | 1 | 53.91 |
| 591 | IPR002429 | 22 | 1.9.3.1 | 22 | 1 | 53.91 |
| 592 | IPR000298 | 17 | 1.9.3.1 | 17 | 1 | 53.91 |
| 593 | IPR004203 | 5 | 1.9.3.1 | 5 | 1 | 53.91 |
| 595 | IPR000883 | 14 | 1.9.3.1 | 13 | 0.93 | 50.06 |
| 596 | IPR007120,IPR007641,IPR007645 | 11 | 2.7.7.6 | 11 | 1 | 47.61 |
| 597 | IPR007641,IPR007645 | 11 | 2.7.7.6 | 11 | 1 | 47.61 |
| 598 | IPR007120,IPR007645 | 11 | 2.7.7.6 | 11 | 1 | 47.61 |
| 599 | IPR007120,IPR007641 | 11 | 2.7.7.6 | 11 | 1 | 47.61 |
| 600 | IPR007645 | 11 | 2.7.7.6 | 11 | 1 | 47.61 |
| 601 | IPR007641 | 11 | 2.7.7.6 | 11 | 1 | 47.61 |
| 602 | IPR007120 | 11 | 2.7.7.6 | 11 | 1 | 47.61 |
| 603 | IPR007120,IPR007641,IPR007642,IPR007644,IPR007645 | 10 | 2.7.7.6 | 10 | 1 | 47.61 |
| 604 | IPR007641,IPR007642,IPR007644,IPR007645 | 10 | 2.7.7.6 | 10 | 1 | 47.61 |
| 605 | IPR007120,IPR007642,IPR007644,IPR007645 | 10 | 2.7.7.6 | 10 | 1 | 47.61 |
| 606 | IPR007120,IPR007641,IPR007644,IPR007645 | 10 | 2.7.7.6 | 10 | 1 | 47.61 |
| 607 | IPR007120,IPR007641,IPR007642,IPR007645 | 10 | 2.7.7.6 | 10 | 1 | 47.61 |
| 608 | IPR007120,IPR007641,IPR007642,IPR007644 | 10 | 2.7.7.6 | 10 | 1 | 47.61 |
| 609 | IPR007642,IPR007644,IPR007645 | 10 | 2.7.7.6 | 10 | 1 | 47.61 |
| 610 | IPR007641,IPR007644,IPR007645 | 10 | 2.7.7.6 | 10 | 1 | 47.61 |
| 611 | IPR007641,IPR007642,IPR007645 | 10 | 2.7.7.6 | 10 | 1 | 47.61 |
| 612 | IPR007641,IPR007642,IPR007644 | 10 | 2.7.7.6 | 10 | 1 | 47.61 |
| 613 | IPR007120,IPR007644,IPR007645 | 10 | 2.7.7.6 | 10 | 1 | 47.61 |
| 614 | IPR007120,IPR007642,IPR007645 | 10 | 2.7.7.6 | 10 | 1 | 47.61 |
| 615 | IPR007120,IPR007642,IPR007644 | 10 | 2.7.7.6 | 10 | 1 | 47.61 |
| 616 | IPR007120,IPR007641,IPR007644 | 10 | 2.7.7.6 | 10 | 1 | 47.61 |
| 617 | IPR007120,IPR007641,IPR007642 | 10 | 2.7.7.6 | 10 | 1 | 47.61 |
| 618 | IPR007644,IPR007645 | 10 | 2.7.7.6 | 10 | 1 | 47.61 |
| 619 | IPR007642,IPR007645 | 10 | 2.7.7.6 | 10 | 1 | 47.61 |
| 620 | IPR007642,IPR007644 | 10 | 2.7.7.6 | 10 | 1 | 47.61 |
| 621 | IPR007641,IPR007644 | 10 | 2.7.7.6 | 10 | 1 | 47.61 |
| 622 | IPR007641,IPR007642 | 10 | 2.7.7.6 | 10 | 1 | 47.61 |
| 623 | IPR007120,IPR007644 | 10 | 2.7.7.6 | 10 | 1 | 47.61 |
| 624 | IPR007120,IPR007642 | 10 | 2.7.7.6 | 10 | 1 | 47.61 |
| 625 | IPR007644 | 10 | 2.7.7.6 | 10 | 1 | 47.61 |
| 626 | IPR007642 | 10 | 2.7.7.6 | 10 | 1 | 47.61 |
| 627 | IPR000722 | 10 | 2.7.7.6 | 10 | 1 | 47.61 |
| 628 | IPR007120,IPR007641,IPR007642,IPR007644,IPR007645,IPR007647 | 9 | 2.7.7.6 | 9 | 1 | 47.61 |
| 629 | IPR007641,IPR007642,IPR007644,IPR007645,IPR007647 | 9 | 2.7.7.6 | 9 | 1 | 47.61 |
| 630 | IPR007120,IPR007642,IPR007644,IPR007645,IPR007647 | 9 | 2.7.7.6 | 9 | 1 | 47.61 |
| 631 | IPR007120,IPR007641,IPR007644,IPR007645,IPR007647 | 9 | 2.7.7.6 | 9 | 1 | 47.61 |
| 632 | IPR007120,IPR007641,IPR007642,IPR007645,IPR007647 | 9 | 2.7.7.6 | 9 | 1 | 47.61 |
| 633 | IPR007120,IPR007641,IPR007642,IPR007644,IPR007647 | 9 | 2.7.7.6 | 9 | 1 | 47.61 |
| 634 | IPR000722,IPR007066,IPR007080,IPR007081,IPR007083 | 9 | 2.7.7.6 | 9 | 1 | 47.61 |
| 635 | IPR007642,IPR007644,IPR007645,IPR007647 | 9 | 2.7.7.6 | 9 | 1 | 47.61 |
| 636 | IPR007641,IPR007644,IPR007645,IPR007647 | 9 | 2.7.7.6 | 9 | 1 | 47.61 |
| 637 | IPR007641,IPR007642,IPR007645,IPR007647 | 9 | 2.7.7.6 | 9 | 1 | 47.61 |
| 638 | IPR007641,IPR007642,IPR007644,IPR007647 | 9 | 2.7.7.6 | 9 | 1 | 47.61 |
| 639 | IPR007120,IPR007644,IPR007645,IPR007647 | 9 | 2.7.7.6 | 9 | 1 | 47.61 |
| 640 | IPR007120,IPR007642,IPR007645,IPR007647 | 9 | 2.7.7.6 | 9 | 1 | 47.61 |
| 641 | IPR007120,IPR007642,IPR007644,IPR007647 | 9 | 2.7.7.6 | 9 | 1 | 47.61 |
| 642 | IPR007120,IPR007641,IPR007645,IPR007647 | 9 | 2.7.7.6 | 9 | 1 | 47.61 |
| 643 | IPR007120,IPR007641,IPR007644,IPR007647 | 9 | 2.7.7.6 | 9 | 1 | 47.61 |
| 644 | IPR007120,IPR007641,IPR007642,IPR007647 | 9 | 2.7.7.6 | 9 | 1 | 47.61 |
| 645 | IPR007066,IPR007080,IPR007081,IPR007083 | 9 | 2.7.7.6 | 9 | 1 | 47.61 |
| 646 | IPR000722,IPR007080,IPR007081,IPR007083 | 9 | 2.7.7.6 | 9 | 1 | 47.61 |
| 647 | IPR000722,IPR007066,IPR007081,IPR007083 | 9 | 2.7.7.6 | 9 | 1 | 47.61 |
| 648 | IPR000722,IPR007066,IPR007080,IPR007083 | 9 | 2.7.7.6 | 9 | 1 | 47.61 |
| 649 | IPR000722,IPR007066,IPR007080,IPR007081 | 9 | 2.7.7.6 | 9 | 1 | 47.61 |
| 650 | IPR007644,IPR007645,IPR007647 | 9 | 2.7.7.6 | 9 | 1 | 47.61 |
| 651 | IPR007642,IPR007645,IPR007647 | 9 | 2.7.7.6 | 9 | 1 | 47.61 |
| 652 | IPR007642,IPR007644,IPR007647 | 9 | 2.7.7.6 | 9 | 1 | 47.61 |
| 653 | IPR007641,IPR007645,IPR007647 | 9 | 2.7.7.6 | 9 | 1 | 47.61 |
| 654 | IPR007641,IPR007644,IPR007647 | 9 | 2.7.7.6 | 9 | 1 | 47.61 |
| 655 | IPR007641,IPR007642,IPR007647 | 9 | 2.7.7.6 | 9 | 1 | 47.61 |
| 656 | IPR007120,IPR007645,IPR007647 | 9 | 2.7.7.6 | 9 | 1 | 47.61 |
| 657 | IPR007120,IPR007644,IPR007647 | 9 | 2.7.7.6 | 9 | 1 | 47.61 |
| 658 | IPR007120,IPR007642,IPR007647 | 9 | 2.7.7.6 | 9 | 1 | 47.61 |
| 659 | IPR007120,IPR007641,IPR007647 | 9 | 2.7.7.6 | 9 | 1 | 47.61 |
| 660 | IPR007080,IPR007081,IPR007083 | 9 | 2.7.7.6 | 9 | 1 | 47.61 |
| 661 | IPR007066,IPR007081,IPR007083 | 9 | 2.7.7.6 | 9 | 1 | 47.61 |
| 662 | IPR007066,IPR007080,IPR007083 | 9 | 2.7.7.6 | 9 | 1 | 47.61 |
| 663 | IPR007066,IPR007080,IPR007081 | 9 | 2.7.7.6 | 9 | 1 | 47.61 |
| 664 | IPR000722,IPR007081,IPR007083 | 9 | 2.7.7.6 | 9 | 1 | 47.61 |
| 665 | IPR000722,IPR007080,IPR007083 | 9 | 2.7.7.6 | 9 | 1 | 47.61 |
| 666 | IPR000722,IPR007080,IPR007081 | 9 | 2.7.7.6 | 9 | 1 | 47.61 |
| 667 | IPR000722,IPR007066,IPR007083 | 9 | 2.7.7.6 | 9 | 1 | 47.61 |
| 668 | IPR000722,IPR007066,IPR007081 | 9 | 2.7.7.6 | 9 | 1 | 47.61 |
| 669 | IPR000722,IPR007066,IPR007080 | 9 | 2.7.7.6 | 9 | 1 | 47.61 |
| 670 | IPR007645,IPR007647 | 9 | 2.7.7.6 | 9 | 1 | 47.61 |
| 671 | IPR007644,IPR007647 | 9 | 2.7.7.6 | 9 | 1 | 47.61 |
| 672 | IPR007642,IPR007647 | 9 | 2.7.7.6 | 9 | 1 | 47.61 |
| 673 | IPR007641,IPR007647 | 9 | 2.7.7.6 | 9 | 1 | 47.61 |
| 674 | IPR007120,IPR007647 | 9 | 2.7.7.6 | 9 | 1 | 47.61 |
| 675 | IPR007081,IPR007083 | 9 | 2.7.7.6 | 9 | 1 | 47.61 |
| 676 | IPR007080,IPR007083 | 9 | 2.7.7.6 | 9 | 1 | 47.61 |
| 677 | IPR007080,IPR007081 | 9 | 2.7.7.6 | 9 | 1 | 47.61 |
| 678 | IPR007066,IPR007083 | 9 | 2.7.7.6 | 9 | 1 | 47.61 |
| 679 | IPR007066,IPR007081 | 9 | 2.7.7.6 | 9 | 1 | 47.61 |
| 680 | IPR007066,IPR007080 | 9 | 2.7.7.6 | 9 | 1 | 47.61 |
| 681 | IPR000722,IPR007083 | 9 | 2.7.7.6 | 9 | 1 | 47.61 |
| 682 | IPR000722,IPR007081 | 9 | 2.7.7.6 | 9 | 1 | 47.61 |
| 683 | IPR000722,IPR007080 | 9 | 2.7.7.6 | 9 | 1 | 47.61 |
| 684 | IPR000722,IPR007066 | 9 | 2.7.7.6 | 9 | 1 | 47.61 |
| 685 | IPR007647 | 9 | 2.7.7.6 | 9 | 1 | 47.61 |
| 686 | IPR007083 | 9 | 2.7.7.6 | 9 | 1 | 47.61 |
| 687 | IPR007081 | 9 | 2.7.7.6 | 9 | 1 | 47.61 |
| 688 | IPR007080 | 9 | 2.7.7.6 | 9 | 1 | 47.61 |
| 689 | IPR007066 | 9 | 2.7.7.6 | 9 | 1 | 47.61 |
| 690 | IPR002092 | 9 | 2.7.7.6 | 9 | 1 | 47.61 |
| 691 | IPR001529 | 9 | 2.7.7.6 | 9 | 1 | 47.61 |
| 692 | IPR000783,IPR005571 | 8 | 2.7.7.6 | 8 | 1 | 47.61 |
| 693 | IPR011261 | 8 | 2.7.7.6 | 8 | 1 | 47.61 |
| 694 | IPR005571 | 8 | 2.7.7.6 | 8 | 1 | 47.61 |
| 695 | IPR000783 | 8 | 2.7.7.6 | 8 | 1 | 47.61 |
| 696 | IPR001222,IPR001529 | 7 | 2.7.7.6 | 7 | 1 | 47.61 |
| 697 | IPR001222 | 7 | 2.7.7.6 | 7 | 1 | 47.61 |
| 698 | IPR007120,IPR007641,IPR007642,IPR007644,IPR007645,IPR007646,IPR007647 | 6 | 2.7.7.6 | 6 | 1 | 47.61 |
| 699 | IPR007641,IPR007642,IPR007644,IPR007645,IPR007646,IPR007647 | 6 | 2.7.7.6 | 6 | 1 | 47.61 |
| 700 | IPR007120,IPR007642,IPR007644,IPR007645,IPR007646,IPR007647 | 6 | 2.7.7.6 | 6 | 1 | 47.61 |
| 701 | IPR007120,IPR007641,IPR007644,IPR007645,IPR007646,IPR007647 | 6 | 2.7.7.6 | 6 | 1 | 47.61 |
| 702 | IPR007120,IPR007641,IPR007642,IPR007645,IPR007646,IPR007647 | 6 | 2.7.7.6 | 6 | 1 | 47.61 |
| 703 | IPR007120,IPR007641,IPR007642,IPR007644,IPR007646,IPR007647 | 6 | 2.7.7.6 | 6 | 1 | 47.61 |
| 704 | IPR007120,IPR007641,IPR007642,IPR007644,IPR007645,IPR007646 | 6 | 2.7.7.6 | 6 | 1 | 47.61 |
| 705 | IPR007642,IPR007644,IPR007645,IPR007646,IPR007647 | 6 | 2.7.7.6 | 6 | 1 | 47.61 |
| 706 | IPR007641,IPR007644,IPR007645,IPR007646,IPR007647 | 6 | 2.7.7.6 | 6 | 1 | 47.61 |
| 707 | IPR007641,IPR007642,IPR007645,IPR007646,IPR007647 | 6 | 2.7.7.6 | 6 | 1 | 47.61 |
| 708 | IPR007641,IPR007642,IPR007644,IPR007646,IPR007647 | 6 | 2.7.7.6 | 6 | 1 | 47.61 |
| 709 | IPR007641,IPR007642,IPR007644,IPR007645,IPR007646 | 6 | 2.7.7.6 | 6 | 1 | 47.61 |
| 710 | IPR007120,IPR007644,IPR007645,IPR007646,IPR007647 | 6 | 2.7.7.6 | 6 | 1 | 47.61 |
| 711 | IPR007120,IPR007642,IPR007645,IPR007646,IPR007647 | 6 | 2.7.7.6 | 6 | 1 | 47.61 |
| 712 | IPR007120,IPR007642,IPR007644,IPR007646,IPR007647 | 6 | 2.7.7.6 | 6 | 1 | 47.61 |
| 713 | IPR007120,IPR007642,IPR007644,IPR007645,IPR007646 | 6 | 2.7.7.6 | 6 | 1 | 47.61 |
| 714 | IPR007120,IPR007641,IPR007645,IPR007646,IPR007647 | 6 | 2.7.7.6 | 6 | 1 | 47.61 |
| 715 | IPR007120,IPR007641,IPR007644,IPR007646,IPR007647 | 6 | 2.7.7.6 | 6 | 1 | 47.61 |
| 716 | IPR007120,IPR007641,IPR007644,IPR007645,IPR007646 | 6 | 2.7.7.6 | 6 | 1 | 47.61 |
| 717 | IPR007120,IPR007641,IPR007642,IPR007646,IPR007647 | 6 | 2.7.7.6 | 6 | 1 | 47.61 |
| 718 | IPR007120,IPR007641,IPR007642,IPR007645,IPR007646 | 6 | 2.7.7.6 | 6 | 1 | 47.61 |
| 719 | IPR007120,IPR007641,IPR007642,IPR007644,IPR007646 | 6 | 2.7.7.6 | 6 | 1 | 47.61 |
| 720 | IPR007644,IPR007645,IPR007646,IPR007647 | 6 | 2.7.7.6 | 6 | 1 | 47.61 |
| 721 | IPR007642,IPR007645,IPR007646,IPR007647 | 6 | 2.7.7.6 | 6 | 1 | 47.61 |
| 722 | IPR007642,IPR007644,IPR007646,IPR007647 | 6 | 2.7.7.6 | 6 | 1 | 47.61 |
| 723 | IPR007642,IPR007644,IPR007645,IPR007646 | 6 | 2.7.7.6 | 6 | 1 | 47.61 |
| 724 | IPR007641,IPR007645,IPR007646,IPR007647 | 6 | 2.7.7.6 | 6 | 1 | 47.61 |
| 725 | IPR007641,IPR007644,IPR007646,IPR007647 | 6 | 2.7.7.6 | 6 | 1 | 47.61 |
| 726 | IPR007641,IPR007644,IPR007645,IPR007646 | 6 | 2.7.7.6 | 6 | 1 | 47.61 |
| 727 | IPR007641,IPR007642,IPR007646,IPR007647 | 6 | 2.7.7.6 | 6 | 1 | 47.61 |
| 728 | IPR007641,IPR007642,IPR007645,IPR007646 | 6 | 2.7.7.6 | 6 | 1 | 47.61 |
| 729 | IPR007641,IPR007642,IPR007644,IPR007646 | 6 | 2.7.7.6 | 6 | 1 | 47.61 |
| 730 | IPR007120,IPR007645,IPR007646,IPR007647 | 6 | 2.7.7.6 | 6 | 1 | 47.61 |
| 731 | IPR007120,IPR007644,IPR007646,IPR007647 | 6 | 2.7.7.6 | 6 | 1 | 47.61 |
| 732 | IPR007120,IPR007644,IPR007645,IPR007646 | 6 | 2.7.7.6 | 6 | 1 | 47.61 |
| 733 | IPR007120,IPR007642,IPR007646,IPR007647 | 6 | 2.7.7.6 | 6 | 1 | 47.61 |
| 734 | IPR007120,IPR007642,IPR007645,IPR007646 | 6 | 2.7.7.6 | 6 | 1 | 47.61 |
| 735 | IPR007120,IPR007642,IPR007644,IPR007646 | 6 | 2.7.7.6 | 6 | 1 | 47.61 |
| 736 | IPR007120,IPR007641,IPR007646,IPR007647 | 6 | 2.7.7.6 | 6 | 1 | 47.61 |
| 737 | IPR007120,IPR007641,IPR007645,IPR007646 | 6 | 2.7.7.6 | 6 | 1 | 47.61 |
| 738 | IPR007120,IPR007641,IPR007644,IPR007646 | 6 | 2.7.7.6 | 6 | 1 | 47.61 |
| 739 | IPR007120,IPR007641,IPR007642,IPR007646 | 6 | 2.7.7.6 | 6 | 1 | 47.61 |
| 740 | IPR007645,IPR007646,IPR007647 | 6 | 2.7.7.6 | 6 | 1 | 47.61 |
| 741 | IPR007644,IPR007646,IPR007647 | 6 | 2.7.7.6 | 6 | 1 | 47.61 |
| 742 | IPR007644,IPR007645,IPR007646 | 6 | 2.7.7.6 | 6 | 1 | 47.61 |
| 743 | IPR007642,IPR007646,IPR007647 | 6 | 2.7.7.6 | 6 | 1 | 47.61 |
| 744 | IPR007642,IPR007645,IPR007646 | 6 | 2.7.7.6 | 6 | 1 | 47.61 |
| 745 | IPR007642,IPR007644,IPR007646 | 6 | 2.7.7.6 | 6 | 1 | 47.61 |
| 746 | IPR007641,IPR007646,IPR007647 | 6 | 2.7.7.6 | 6 | 1 | 47.61 |
| 747 | IPR007641,IPR007645,IPR007646 | 6 | 2.7.7.6 | 6 | 1 | 47.61 |
| 748 | IPR007641,IPR007644,IPR007646 | 6 | 2.7.7.6 | 6 | 1 | 47.61 |
| 749 | IPR007641,IPR007642,IPR007646 | 6 | 2.7.7.6 | 6 | 1 | 47.61 |
| 750 | IPR007120,IPR007646,IPR007647 | 6 | 2.7.7.6 | 6 | 1 | 47.61 |
| 751 | IPR007120,IPR007645,IPR007646 | 6 | 2.7.7.6 | 6 | 1 | 47.61 |
| 752 | IPR007120,IPR007644,IPR007646 | 6 | 2.7.7.6 | 6 | 1 | 47.61 |
| 753 | IPR007120,IPR007642,IPR007646 | 6 | 2.7.7.6 | 6 | 1 | 47.61 |
| 754 | IPR007120,IPR007641,IPR007646 | 6 | 2.7.7.6 | 6 | 1 | 47.61 |
| 755 | IPR007646,IPR007647 | 6 | 2.7.7.6 | 6 | 1 | 47.61 |
| 756 | IPR007645,IPR007646 | 6 | 2.7.7.6 | 6 | 1 | 47.61 |
| 757 | IPR007644,IPR007646 | 6 | 2.7.7.6 | 6 | 1 | 47.61 |
| 758 | IPR007642,IPR007646 | 6 | 2.7.7.6 | 6 | 1 | 47.61 |
| 759 | IPR007641,IPR007646 | 6 | 2.7.7.6 | 6 | 1 | 47.61 |
| 760 | IPR007120,IPR007646 | 6 | 2.7.7.6 | 6 | 1 | 47.61 |
| 761 | IPR007646 | 6 | 2.7.7.6 | 6 | 1 | 47.61 |
| 762 | IPR000684,IPR000722,IPR007066,IPR007073,IPR007075,IPR007080,IPR007081,IPR007083 | 4 | 2.7.7.6 | 4 | 1 | 47.61 |
| 763 | IPR000722,IPR007066,IPR007073,IPR007075,IPR007080,IPR007081,IPR007083 | 4 | 2.7.7.6 | 4 | 1 | 47.61 |
| 764 | IPR000684,IPR007066,IPR007073,IPR007075,IPR007080,IPR007081,IPR007083 | 4 | 2.7.7.6 | 4 | 1 | 47.61 |
| 765 | IPR000684,IPR000722,IPR007073,IPR007075,IPR007080,IPR007081,IPR007083 | 4 | 2.7.7.6 | 4 | 1 | 47.61 |
| 766 | IPR000684,IPR000722,IPR007066,IPR007075,IPR007080,IPR007081,IPR007083 | 4 | 2.7.7.6 | 4 | 1 | 47.61 |
| 767 | IPR000684,IPR000722,IPR007066,IPR007073,IPR007080,IPR007081,IPR007083 | 4 | 2.7.7.6 | 4 | 1 | 47.61 |
| 768 | IPR000684,IPR000722,IPR007066,IPR007073,IPR007075,IPR007081,IPR007083 | 4 | 2.7.7.6 | 4 | 1 | 47.61 |
| 769 | IPR000684,IPR000722,IPR007066,IPR007073,IPR007075,IPR007080,IPR007083 | 4 | 2.7.7.6 | 4 | 1 | 47.61 |
| 770 | IPR000684,IPR000722,IPR007066,IPR007073,IPR007075,IPR007080,IPR007081 | 4 | 2.7.7.6 | 4 | 1 | 47.61 |
| 771 | IPR007120,IPR007641,IPR007642,IPR007644,IPR007645,IPR009674 | 4 | 2.7.7.6 | 4 | 1 | 47.61 |
| 772 | IPR007066,IPR007073,IPR007075,IPR007080,IPR007081,IPR007083 | 4 | 2.7.7.6 | 4 | 1 | 47.61 |
| 773 | IPR000722,IPR007073,IPR007075,IPR007080,IPR007081,IPR007083 | 4 | 2.7.7.6 | 4 | 1 | 47.61 |
| 774 | IPR000722,IPR007066,IPR007075,IPR007080,IPR007081,IPR007083 | 4 | 2.7.7.6 | 4 | 1 | 47.61 |
| 775 | IPR000722,IPR007066,IPR007073,IPR007080,IPR007081,IPR007083 | 4 | 2.7.7.6 | 4 | 1 | 47.61 |
| 776 | IPR000722,IPR007066,IPR007073,IPR007075,IPR007081,IPR007083 | 4 | 2.7.7.6 | 4 | 1 | 47.61 |
| 777 | IPR000722,IPR007066,IPR007073,IPR007075,IPR007080,IPR007083 | 4 | 2.7.7.6 | 4 | 1 | 47.61 |
| 778 | IPR000722,IPR007066,IPR007073,IPR007075,IPR007080,IPR007081 | 4 | 2.7.7.6 | 4 | 1 | 47.61 |
| 779 | IPR000684,IPR007073,IPR007075,IPR007080,IPR007081,IPR007083 | 4 | 2.7.7.6 | 4 | 1 | 47.61 |
| 780 | IPR000684,IPR007066,IPR007075,IPR007080,IPR007081,IPR007083 | 4 | 2.7.7.6 | 4 | 1 | 47.61 |
| 781 | IPR000684,IPR007066,IPR007073,IPR007080,IPR007081,IPR007083 | 4 | 2.7.7.6 | 4 | 1 | 47.61 |
| 782 | IPR000684,IPR007066,IPR007073,IPR007075,IPR007081,IPR007083 | 4 | 2.7.7.6 | 4 | 1 | 47.61 |
| 783 | IPR000684,IPR007066,IPR007073,IPR007075,IPR007080,IPR007083 | 4 | 2.7.7.6 | 4 | 1 | 47.61 |
| 784 | IPR000684,IPR007066,IPR007073,IPR007075,IPR007080,IPR007081 | 4 | 2.7.7.6 | 4 | 1 | 47.61 |
| 785 | IPR000684,IPR000722,IPR007075,IPR007080,IPR007081,IPR007083 | 4 | 2.7.7.6 | 4 | 1 | 47.61 |
| 786 | IPR000684,IPR000722,IPR007073,IPR007080,IPR007081,IPR007083 | 4 | 2.7.7.6 | 4 | 1 | 47.61 |
| 787 | IPR000684,IPR000722,IPR007073,IPR007075,IPR007081,IPR007083 | 4 | 2.7.7.6 | 4 | 1 | 47.61 |
| 788 | IPR000684,IPR000722,IPR007073,IPR007075,IPR007080,IPR007083 | 4 | 2.7.7.6 | 4 | 1 | 47.61 |
| 789 | IPR000684,IPR000722,IPR007073,IPR007075,IPR007080,IPR007081 | 4 | 2.7.7.6 | 4 | 1 | 47.61 |
| 790 | IPR000684,IPR000722,IPR007066,IPR007080,IPR007081,IPR007083 | 4 | 2.7.7.6 | 4 | 1 | 47.61 |
| 791 | IPR000684,IPR000722,IPR007066,IPR007075,IPR007081,IPR007083 | 4 | 2.7.7.6 | 4 | 1 | 47.61 |
| 792 | IPR000684,IPR000722,IPR007066,IPR007075,IPR007080,IPR007083 | 4 | 2.7.7.6 | 4 | 1 | 47.61 |
| 793 | IPR000684,IPR000722,IPR007066,IPR007075,IPR007080,IPR007081 | 4 | 2.7.7.6 | 4 | 1 | 47.61 |
| 794 | IPR000684,IPR000722,IPR007066,IPR007073,IPR007081,IPR007083 | 4 | 2.7.7.6 | 4 | 1 | 47.61 |
| 795 | IPR000684,IPR000722,IPR007066,IPR007073,IPR007080,IPR007083 | 4 | 2.7.7.6 | 4 | 1 | 47.61 |
| 796 | IPR000684,IPR000722,IPR007066,IPR007073,IPR007080,IPR007081 | 4 | 2.7.7.6 | 4 | 1 | 47.61 |
| 797 | IPR000684,IPR000722,IPR007066,IPR007073,IPR007075,IPR007083 | 4 | 2.7.7.6 | 4 | 1 | 47.61 |
| 798 | IPR000684,IPR000722,IPR007066,IPR007073,IPR007075,IPR007081 | 4 | 2.7.7.6 | 4 | 1 | 47.61 |
| 799 | IPR000684,IPR000722,IPR007066,IPR007073,IPR007075,IPR007080 | 4 | 2.7.7.6 | 4 | 1 | 47.61 |
| 800 | IPR007641,IPR007642,IPR007644,IPR007645,IPR009674 | 4 | 2.7.7.6 | 4 | 1 | 47.61 |
| 801 | IPR007120,IPR007642,IPR007644,IPR007645,IPR009674 | 4 | 2.7.7.6 | 4 | 1 | 47.61 |
| 802 | IPR007120,IPR007641,IPR007644,IPR007645,IPR009674 | 4 | 2.7.7.6 | 4 | 1 | 47.61 |
| 803 | IPR007120,IPR007641,IPR007642,IPR007645,IPR009674 | 4 | 2.7.7.6 | 4 | 1 | 47.61 |
| 804 | IPR007120,IPR007641,IPR007642,IPR007644,IPR009674 | 4 | 2.7.7.6 | 4 | 1 | 47.61 |
| 805 | IPR007073,IPR007075,IPR007080,IPR007081,IPR007083 | 4 | 2.7.7.6 | 4 | 1 | 47.61 |
| 806 | IPR007066,IPR007075,IPR007080,IPR007081,IPR007083 | 4 | 2.7.7.6 | 4 | 1 | 47.61 |
| 807 | IPR007066,IPR007073,IPR007080,IPR007081,IPR007083 | 4 | 2.7.7.6 | 4 | 1 | 47.61 |
| 808 | IPR007066,IPR007073,IPR007075,IPR007081,IPR007083 | 4 | 2.7.7.6 | 4 | 1 | 47.61 |
| 809 | IPR007066,IPR007073,IPR007075,IPR007080,IPR007083 | 4 | 2.7.7.6 | 4 | 1 | 47.61 |
| 810 | IPR007066,IPR007073,IPR007075,IPR007080,IPR007081 | 4 | 2.7.7.6 | 4 | 1 | 47.61 |
| 811 | IPR000722,IPR007075,IPR007080,IPR007081,IPR007083 | 4 | 2.7.7.6 | 4 | 1 | 47.61 |
| 812 | IPR000722,IPR007073,IPR007080,IPR007081,IPR007083 | 4 | 2.7.7.6 | 4 | 1 | 47.61 |
| 813 | IPR000722,IPR007073,IPR007075,IPR007081,IPR007083 | 4 | 2.7.7.6 | 4 | 1 | 47.61 |
| 814 | IPR000722,IPR007073,IPR007075,IPR007080,IPR007083 | 4 | 2.7.7.6 | 4 | 1 | 47.61 |
| 815 | IPR000722,IPR007073,IPR007075,IPR007080,IPR007081 | 4 | 2.7.7.6 | 4 | 1 | 47.61 |
| 816 | IPR000722,IPR007066,IPR007075,IPR007081,IPR007083 | 4 | 2.7.7.6 | 4 | 1 | 47.61 |
| 817 | IPR000722,IPR007066,IPR007075,IPR007080,IPR007083 | 4 | 2.7.7.6 | 4 | 1 | 47.61 |
| 818 | IPR000722,IPR007066,IPR007075,IPR007080,IPR007081 | 4 | 2.7.7.6 | 4 | 1 | 47.61 |
| 819 | IPR000722,IPR007066,IPR007073,IPR007081,IPR007083 | 4 | 2.7.7.6 | 4 | 1 | 47.61 |
| 820 | IPR000722,IPR007066,IPR007073,IPR007080,IPR007083 | 4 | 2.7.7.6 | 4 | 1 | 47.61 |
| 821 | IPR000722,IPR007066,IPR007073,IPR007080,IPR007081 | 4 | 2.7.7.6 | 4 | 1 | 47.61 |
| 822 | IPR000722,IPR007066,IPR007073,IPR007075,IPR007083 | 4 | 2.7.7.6 | 4 | 1 | 47.61 |
| 823 | IPR000722,IPR007066,IPR007073,IPR007075,IPR007081 | 4 | 2.7.7.6 | 4 | 1 | 47.61 |
| 824 | IPR000722,IPR007066,IPR007073,IPR007075,IPR007080 | 4 | 2.7.7.6 | 4 | 1 | 47.61 |
| 825 | IPR000684,IPR007075,IPR007080,IPR007081,IPR007083 | 4 | 2.7.7.6 | 4 | 1 | 47.61 |
| 826 | IPR000684,IPR007073,IPR007080,IPR007081,IPR007083 | 4 | 2.7.7.6 | 4 | 1 | 47.61 |
| 827 | IPR000684,IPR007073,IPR007075,IPR007081,IPR007083 | 4 | 2.7.7.6 | 4 | 1 | 47.61 |
| 828 | IPR000684,IPR007073,IPR007075,IPR007080,IPR007083 | 4 | 2.7.7.6 | 4 | 1 | 47.61 |
| 829 | IPR000684,IPR007073,IPR007075,IPR007080,IPR007081 | 4 | 2.7.7.6 | 4 | 1 | 47.61 |
| 830 | IPR000684,IPR007066,IPR007080,IPR007081,IPR007083 | 4 | 2.7.7.6 | 4 | 1 | 47.61 |
| 831 | IPR000684,IPR007066,IPR007075,IPR007081,IPR007083 | 4 | 2.7.7.6 | 4 | 1 | 47.61 |
| 832 | IPR000684,IPR007066,IPR007075,IPR007080,IPR007083 | 4 | 2.7.7.6 | 4 | 1 | 47.61 |
| 833 | IPR000684,IPR007066,IPR007075,IPR007080,IPR007081 | 4 | 2.7.7.6 | 4 | 1 | 47.61 |
| 834 | IPR000684,IPR007066,IPR007073,IPR007081,IPR007083 | 4 | 2.7.7.6 | 4 | 1 | 47.61 |
| 835 | IPR000684,IPR007066,IPR007073,IPR007080,IPR007083 | 4 | 2.7.7.6 | 4 | 1 | 47.61 |
| 836 | IPR000684,IPR007066,IPR007073,IPR007080,IPR007081 | 4 | 2.7.7.6 | 4 | 1 | 47.61 |
| 837 | IPR000684,IPR007066,IPR007073,IPR007075,IPR007083 | 4 | 2.7.7.6 | 4 | 1 | 47.61 |
| 838 | IPR000684,IPR007066,IPR007073,IPR007075,IPR007081 | 4 | 2.7.7.6 | 4 | 1 | 47.61 |
| 839 | IPR000684,IPR007066,IPR007073,IPR007075,IPR007080 | 4 | 2.7.7.6 | 4 | 1 | 47.61 |
| 840 | IPR000684,IPR000722,IPR007080,IPR007081,IPR007083 | 4 | 2.7.7.6 | 4 | 1 | 47.61 |
| 841 | IPR000684,IPR000722,IPR007075,IPR007081,IPR007083 | 4 | 2.7.7.6 | 4 | 1 | 47.61 |
| 842 | IPR000684,IPR000722,IPR007075,IPR007080,IPR007083 | 4 | 2.7.7.6 | 4 | 1 | 47.61 |
| 843 | IPR000684,IPR000722,IPR007075,IPR007080,IPR007081 | 4 | 2.7.7.6 | 4 | 1 | 47.61 |
| 844 | IPR000684,IPR000722,IPR007073,IPR007081,IPR007083 | 4 | 2.7.7.6 | 4 | 1 | 47.61 |
| 845 | IPR000684,IPR000722,IPR007073,IPR007080,IPR007083 | 4 | 2.7.7.6 | 4 | 1 | 47.61 |
| 846 | IPR000684,IPR000722,IPR007073,IPR007080,IPR007081 | 4 | 2.7.7.6 | 4 | 1 | 47.61 |
| 847 | IPR000684,IPR000722,IPR007073,IPR007075,IPR007083 | 4 | 2.7.7.6 | 4 | 1 | 47.61 |
| 848 | IPR000684,IPR000722,IPR007073,IPR007075,IPR007081 | 4 | 2.7.7.6 | 4 | 1 | 47.61 |
| 849 | IPR000684,IPR000722,IPR007073,IPR007075,IPR007080 | 4 | 2.7.7.6 | 4 | 1 | 47.61 |
| 850 | IPR000684,IPR000722,IPR007066,IPR007081,IPR007083 | 4 | 2.7.7.6 | 4 | 1 | 47.61 |
| 851 | IPR000684,IPR000722,IPR007066,IPR007080,IPR007083 | 4 | 2.7.7.6 | 4 | 1 | 47.61 |
| 852 | IPR000684,IPR000722,IPR007066,IPR007080,IPR007081 | 4 | 2.7.7.6 | 4 | 1 | 47.61 |
| 853 | IPR000684,IPR000722,IPR007066,IPR007075,IPR007083 | 4 | 2.7.7.6 | 4 | 1 | 47.61 |
| 854 | IPR000684,IPR000722,IPR007066,IPR007075,IPR007081 | 4 | 2.7.7.6 | 4 | 1 | 47.61 |
| 855 | IPR000684,IPR000722,IPR007066,IPR007075,IPR007080 | 4 | 2.7.7.6 | 4 | 1 | 47.61 |
| 856 | IPR000684,IPR000722,IPR007066,IPR007073,IPR007083 | 4 | 2.7.7.6 | 4 | 1 | 47.61 |
| 857 | IPR000684,IPR000722,IPR007066,IPR007073,IPR007081 | 4 | 2.7.7.6 | 4 | 1 | 47.61 |
| 858 | IPR000684,IPR000722,IPR007066,IPR007073,IPR007080 | 4 | 2.7.7.6 | 4 | 1 | 47.61 |
| 859 | IPR000684,IPR000722,IPR007066,IPR007073,IPR007075 | 4 | 2.7.7.6 | 4 | 1 | 47.61 |
| 860 | IPR007642,IPR007644,IPR007645,IPR009674 | 4 | 2.7.7.6 | 4 | 1 | 47.61 |
| 861 | IPR007641,IPR007644,IPR007645,IPR009674 | 4 | 2.7.7.6 | 4 | 1 | 47.61 |
| 862 | IPR007641,IPR007642,IPR007645,IPR009674 | 4 | 2.7.7.6 | 4 | 1 | 47.61 |
| 863 | IPR007641,IPR007642,IPR007644,IPR009674 | 4 | 2.7.7.6 | 4 | 1 | 47.61 |
| 864 | IPR007120,IPR007644,IPR007645,IPR009674 | 4 | 2.7.7.6 | 4 | 1 | 47.61 |
| 865 | IPR007120,IPR007642,IPR007645,IPR009674 | 4 | 2.7.7.6 | 4 | 1 | 47.61 |
| 866 | IPR007120,IPR007642,IPR007644,IPR009674 | 4 | 2.7.7.6 | 4 | 1 | 47.61 |
| 867 | IPR007120,IPR007641,IPR007645,IPR009674 | 4 | 2.7.7.6 | 4 | 1 | 47.61 |
| 868 | IPR007120,IPR007641,IPR007644,IPR009674 | 4 | 2.7.7.6 | 4 | 1 | 47.61 |
| 869 | IPR007120,IPR007641,IPR007642,IPR009674 | 4 | 2.7.7.6 | 4 | 1 | 47.61 |
| 870 | IPR007075,IPR007080,IPR007081,IPR007083 | 4 | 2.7.7.6 | 4 | 1 | 47.61 |
| 871 | IPR007073,IPR007080,IPR007081,IPR007083 | 4 | 2.7.7.6 | 4 | 1 | 47.61 |
| 872 | IPR007073,IPR007075,IPR007081,IPR007083 | 4 | 2.7.7.6 | 4 | 1 | 47.61 |
| 873 | IPR007073,IPR007075,IPR007080,IPR007083 | 4 | 2.7.7.6 | 4 | 1 | 47.61 |
| 874 | IPR007073,IPR007075,IPR007080,IPR007081 | 4 | 2.7.7.6 | 4 | 1 | 47.61 |
| 875 | IPR007066,IPR007075,IPR007081,IPR007083 | 4 | 2.7.7.6 | 4 | 1 | 47.61 |
| 876 | IPR007066,IPR007075,IPR007080,IPR007083 | 4 | 2.7.7.6 | 4 | 1 | 47.61 |
| 877 | IPR007066,IPR007075,IPR007080,IPR007081 | 4 | 2.7.7.6 | 4 | 1 | 47.61 |
| 878 | IPR007066,IPR007073,IPR007081,IPR007083 | 4 | 2.7.7.6 | 4 | 1 | 47.61 |
| 879 | IPR007066,IPR007073,IPR007080,IPR007083 | 4 | 2.7.7.6 | 4 | 1 | 47.61 |
| 880 | IPR007066,IPR007073,IPR007080,IPR007081 | 4 | 2.7.7.6 | 4 | 1 | 47.61 |
| 881 | IPR007066,IPR007073,IPR007075,IPR007083 | 4 | 2.7.7.6 | 4 | 1 | 47.61 |
| 882 | IPR007066,IPR007073,IPR007075,IPR007081 | 4 | 2.7.7.6 | 4 | 1 | 47.61 |
| 883 | IPR007066,IPR007073,IPR007075,IPR007080 | 4 | 2.7.7.6 | 4 | 1 | 47.61 |
| 884 | IPR000722,IPR007075,IPR007081,IPR007083 | 4 | 2.7.7.6 | 4 | 1 | 47.61 |
| 885 | IPR000722,IPR007075,IPR007080,IPR007083 | 4 | 2.7.7.6 | 4 | 1 | 47.61 |
| 886 | IPR000722,IPR007075,IPR007080,IPR007081 | 4 | 2.7.7.6 | 4 | 1 | 47.61 |
| 887 | IPR000722,IPR007073,IPR007081,IPR007083 | 4 | 2.7.7.6 | 4 | 1 | 47.61 |
| 888 | IPR000722,IPR007073,IPR007080,IPR007083 | 4 | 2.7.7.6 | 4 | 1 | 47.61 |
| 889 | IPR000722,IPR007073,IPR007080,IPR007081 | 4 | 2.7.7.6 | 4 | 1 | 47.61 |
| 890 | IPR000722,IPR007073,IPR007075,IPR007083 | 4 | 2.7.7.6 | 4 | 1 | 47.61 |
| 891 | IPR000722,IPR007073,IPR007075,IPR007081 | 4 | 2.7.7.6 | 4 | 1 | 47.61 |
| 892 | IPR000722,IPR007073,IPR007075,IPR007080 | 4 | 2.7.7.6 | 4 | 1 | 47.61 |
| 893 | IPR000722,IPR007066,IPR007075,IPR007083 | 4 | 2.7.7.6 | 4 | 1 | 47.61 |
| 894 | IPR000722,IPR007066,IPR007075,IPR007081 | 4 | 2.7.7.6 | 4 | 1 | 47.61 |
| 895 | IPR000722,IPR007066,IPR007075,IPR007080 | 4 | 2.7.7.6 | 4 | 1 | 47.61 |
| 896 | IPR000722,IPR007066,IPR007073,IPR007083 | 4 | 2.7.7.6 | 4 | 1 | 47.61 |
| 897 | IPR000722,IPR007066,IPR007073,IPR007081 | 4 | 2.7.7.6 | 4 | 1 | 47.61 |
| 898 | IPR000722,IPR007066,IPR007073,IPR007080 | 4 | 2.7.7.6 | 4 | 1 | 47.61 |
| 899 | IPR000722,IPR007066,IPR007073,IPR007075 | 4 | 2.7.7.6 | 4 | 1 | 47.61 |
| 900 | IPR000684,IPR007080,IPR007081,IPR007083 | 4 | 2.7.7.6 | 4 | 1 | 47.61 |
| 901 | IPR000684,IPR007075,IPR007081,IPR007083 | 4 | 2.7.7.6 | 4 | 1 | 47.61 |
| 902 | IPR000684,IPR007075,IPR007080,IPR007083 | 4 | 2.7.7.6 | 4 | 1 | 47.61 |
| 903 | IPR000684,IPR007075,IPR007080,IPR007081 | 4 | 2.7.7.6 | 4 | 1 | 47.61 |
| 904 | IPR000684,IPR007073,IPR007081,IPR007083 | 4 | 2.7.7.6 | 4 | 1 | 47.61 |
| 905 | IPR000684,IPR007073,IPR007080,IPR007083 | 4 | 2.7.7.6 | 4 | 1 | 47.61 |
| 906 | IPR000684,IPR007073,IPR007080,IPR007081 | 4 | 2.7.7.6 | 4 | 1 | 47.61 |
| 907 | IPR000684,IPR007073,IPR007075,IPR007083 | 4 | 2.7.7.6 | 4 | 1 | 47.61 |
| 908 | IPR000684,IPR007073,IPR007075,IPR007081 | 4 | 2.7.7.6 | 4 | 1 | 47.61 |
| 909 | IPR000684,IPR007073,IPR007075,IPR007080 | 4 | 2.7.7.6 | 4 | 1 | 47.61 |
| 910 | IPR000684,IPR007066,IPR007081,IPR007083 | 4 | 2.7.7.6 | 4 | 1 | 47.61 |
| 911 | IPR000684,IPR007066,IPR007080,IPR007083 | 4 | 2.7.7.6 | 4 | 1 | 47.61 |
| 912 | IPR000684,IPR007066,IPR007080,IPR007081 | 4 | 2.7.7.6 | 4 | 1 | 47.61 |
| 913 | IPR000684,IPR007066,IPR007075,IPR007083 | 4 | 2.7.7.6 | 4 | 1 | 47.61 |
| 914 | IPR000684,IPR007066,IPR007075,IPR007081 | 4 | 2.7.7.6 | 4 | 1 | 47.61 |
| 915 | IPR000684,IPR007066,IPR007075,IPR007080 | 4 | 2.7.7.6 | 4 | 1 | 47.61 |
| 916 | IPR000684,IPR007066,IPR007073,IPR007083 | 4 | 2.7.7.6 | 4 | 1 | 47.61 |
| 917 | IPR000684,IPR007066,IPR007073,IPR007081 | 4 | 2.7.7.6 | 4 | 1 | 47.61 |
| 918 | IPR000684,IPR007066,IPR007073,IPR007080 | 4 | 2.7.7.6 | 4 | 1 | 47.61 |
| 919 | IPR000684,IPR007066,IPR007073,IPR007075 | 4 | 2.7.7.6 | 4 | 1 | 47.61 |
| 920 | IPR000684,IPR000722,IPR007081,IPR007083 | 4 | 2.7.7.6 | 4 | 1 | 47.61 |
| 921 | IPR000684,IPR000722,IPR007080,IPR007083 | 4 | 2.7.7.6 | 4 | 1 | 47.61 |
| 922 | IPR000684,IPR000722,IPR007080,IPR007081 | 4 | 2.7.7.6 | 4 | 1 | 47.61 |
| 923 | IPR000684,IPR000722,IPR007075,IPR007083 | 4 | 2.7.7.6 | 4 | 1 | 47.61 |
| 924 | IPR000684,IPR000722,IPR007075,IPR007081 | 4 | 2.7.7.6 | 4 | 1 | 47.61 |
| 925 | IPR000684,IPR000722,IPR007075,IPR007080 | 4 | 2.7.7.6 | 4 | 1 | 47.61 |
| 926 | IPR000684,IPR000722,IPR007073,IPR007083 | 4 | 2.7.7.6 | 4 | 1 | 47.61 |
| 927 | IPR000684,IPR000722,IPR007073,IPR007081 | 4 | 2.7.7.6 | 4 | 1 | 47.61 |
| 928 | IPR000684,IPR000722,IPR007073,IPR007080 | 4 | 2.7.7.6 | 4 | 1 | 47.61 |
| 929 | IPR000684,IPR000722,IPR007073,IPR007075 | 4 | 2.7.7.6 | 4 | 1 | 47.61 |
| 930 | IPR000684,IPR000722,IPR007066,IPR007083 | 4 | 2.7.7.6 | 4 | 1 | 47.61 |
| 931 | IPR000684,IPR000722,IPR007066,IPR007081 | 4 | 2.7.7.6 | 4 | 1 | 47.61 |
| 932 | IPR000684,IPR000722,IPR007066,IPR007080 | 4 | 2.7.7.6 | 4 | 1 | 47.61 |
| 933 | IPR000684,IPR000722,IPR007066,IPR007075 | 4 | 2.7.7.6 | 4 | 1 | 47.61 |
| 934 | IPR000684,IPR000722,IPR007066,IPR007073 | 4 | 2.7.7.6 | 4 | 1 | 47.61 |
| 935 | IPR007644,IPR007645,IPR009674 | 4 | 2.7.7.6 | 4 | 1 | 47.61 |
| 936 | IPR007642,IPR007645,IPR009674 | 4 | 2.7.7.6 | 4 | 1 | 47.61 |
| 937 | IPR007642,IPR007644,IPR009674 | 4 | 2.7.7.6 | 4 | 1 | 47.61 |
| 938 | IPR007641,IPR007645,IPR009674 | 4 | 2.7.7.6 | 4 | 1 | 47.61 |
| 939 | IPR007641,IPR007644,IPR009674 | 4 | 2.7.7.6 | 4 | 1 | 47.61 |
| 940 | IPR007641,IPR007642,IPR009674 | 4 | 2.7.7.6 | 4 | 1 | 47.61 |
| 941 | IPR007120,IPR007645,IPR009674 | 4 | 2.7.7.6 | 4 | 1 | 47.61 |
| 942 | IPR007120,IPR007644,IPR009674 | 4 | 2.7.7.6 | 4 | 1 | 47.61 |
| 943 | IPR007120,IPR007642,IPR009674 | 4 | 2.7.7.6 | 4 | 1 | 47.61 |
| 944 | IPR007120,IPR007641,IPR009674 | 4 | 2.7.7.6 | 4 | 1 | 47.61 |
| 945 | IPR007075,IPR007081,IPR007083 | 4 | 2.7.7.6 | 4 | 1 | 47.61 |
| 946 | IPR007075,IPR007080,IPR007083 | 4 | 2.7.7.6 | 4 | 1 | 47.61 |
| 947 | IPR007075,IPR007080,IPR007081 | 4 | 2.7.7.6 | 4 | 1 | 47.61 |
| 948 | IPR007073,IPR007081,IPR007083 | 4 | 2.7.7.6 | 4 | 1 | 47.61 |
| 949 | IPR007073,IPR007080,IPR007083 | 4 | 2.7.7.6 | 4 | 1 | 47.61 |
| 950 | IPR007073,IPR007080,IPR007081 | 4 | 2.7.7.6 | 4 | 1 | 47.61 |
| 951 | IPR007073,IPR007075,IPR007083 | 4 | 2.7.7.6 | 4 | 1 | 47.61 |
| 952 | IPR007073,IPR007075,IPR007081 | 4 | 2.7.7.6 | 4 | 1 | 47.61 |
| 953 | IPR007073,IPR007075,IPR007080 | 4 | 2.7.7.6 | 4 | 1 | 47.61 |
| 954 | IPR007066,IPR007075,IPR007083 | 4 | 2.7.7.6 | 4 | 1 | 47.61 |
| 955 | IPR007066,IPR007075,IPR007081 | 4 | 2.7.7.6 | 4 | 1 | 47.61 |
| 956 | IPR007066,IPR007075,IPR007080 | 4 | 2.7.7.6 | 4 | 1 | 47.61 |
| 957 | IPR007066,IPR007073,IPR007083 | 4 | 2.7.7.6 | 4 | 1 | 47.61 |
| 958 | IPR007066,IPR007073,IPR007081 | 4 | 2.7.7.6 | 4 | 1 | 47.61 |
| 959 | IPR007066,IPR007073,IPR007080 | 4 | 2.7.7.6 | 4 | 1 | 47.61 |
| 960 | IPR007066,IPR007073,IPR007075 | 4 | 2.7.7.6 | 4 | 1 | 47.61 |
| 961 | IPR000722,IPR007075,IPR007083 | 4 | 2.7.7.6 | 4 | 1 | 47.61 |
| 962 | IPR000722,IPR007075,IPR007081 | 4 | 2.7.7.6 | 4 | 1 | 47.61 |
| 963 | IPR000722,IPR007075,IPR007080 | 4 | 2.7.7.6 | 4 | 1 | 47.61 |
| 964 | IPR000722,IPR007073,IPR007083 | 4 | 2.7.7.6 | 4 | 1 | 47.61 |
| 965 | IPR000722,IPR007073,IPR007081 | 4 | 2.7.7.6 | 4 | 1 | 47.61 |
| 966 | IPR000722,IPR007073,IPR007080 | 4 | 2.7.7.6 | 4 | 1 | 47.61 |
| 967 | IPR000722,IPR007073,IPR007075 | 4 | 2.7.7.6 | 4 | 1 | 47.61 |
| 968 | IPR000722,IPR007066,IPR007075 | 4 | 2.7.7.6 | 4 | 1 | 47.61 |
| 969 | IPR000722,IPR007066,IPR007073 | 4 | 2.7.7.6 | 4 | 1 | 47.61 |
| 970 | IPR000684,IPR007081,IPR007083 | 4 | 2.7.7.6 | 4 | 1 | 47.61 |
| 971 | IPR000684,IPR007080,IPR007083 | 4 | 2.7.7.6 | 4 | 1 | 47.61 |
| 972 | IPR000684,IPR007080,IPR007081 | 4 | 2.7.7.6 | 4 | 1 | 47.61 |
| 973 | IPR000684,IPR007075,IPR007083 | 4 | 2.7.7.6 | 4 | 1 | 47.61 |
| 974 | IPR000684,IPR007075,IPR007081 | 4 | 2.7.7.6 | 4 | 1 | 47.61 |
| 975 | IPR000684,IPR007075,IPR007080 | 4 | 2.7.7.6 | 4 | 1 | 47.61 |
| 976 | IPR000684,IPR007073,IPR007083 | 4 | 2.7.7.6 | 4 | 1 | 47.61 |
| 977 | IPR000684,IPR007073,IPR007081 | 4 | 2.7.7.6 | 4 | 1 | 47.61 |
| 978 | IPR000684,IPR007073,IPR007080 | 4 | 2.7.7.6 | 4 | 1 | 47.61 |
| 979 | IPR000684,IPR007073,IPR007075 | 4 | 2.7.7.6 | 4 | 1 | 47.61 |
| 980 | IPR000684,IPR007066,IPR007083 | 4 | 2.7.7.6 | 4 | 1 | 47.61 |
| 981 | IPR000684,IPR007066,IPR007081 | 4 | 2.7.7.6 | 4 | 1 | 47.61 |
| 982 | IPR000684,IPR007066,IPR007080 | 4 | 2.7.7.6 | 4 | 1 | 47.61 |
| 983 | IPR000684,IPR007066,IPR007075 | 4 | 2.7.7.6 | 4 | 1 | 47.61 |
| 984 | IPR000684,IPR007066,IPR007073 | 4 | 2.7.7.6 | 4 | 1 | 47.61 |
| 985 | IPR000684,IPR000722,IPR007083 | 4 | 2.7.7.6 | 4 | 1 | 47.61 |
| 986 | IPR000684,IPR000722,IPR007081 | 4 | 2.7.7.6 | 4 | 1 | 47.61 |
| 987 | IPR000684,IPR000722,IPR007080 | 4 | 2.7.7.6 | 4 | 1 | 47.61 |
| 988 | IPR000684,IPR000722,IPR007075 | 4 | 2.7.7.6 | 4 | 1 | 47.61 |
| 989 | IPR000684,IPR000722,IPR007073 | 4 | 2.7.7.6 | 4 | 1 | 47.61 |
| 990 | IPR000684,IPR000722,IPR007066 | 4 | 2.7.7.6 | 4 | 1 | 47.61 |
| 991 | IPR011261,IPR011262 | 4 | 2.7.7.6 | 4 | 1 | 47.61 |
| 992 | IPR007645,IPR009674 | 4 | 2.7.7.6 | 4 | 1 | 47.61 |
| 993 | IPR007644,IPR009674 | 4 | 2.7.7.6 | 4 | 1 | 47.61 |
| 994 | IPR007642,IPR009674 | 4 | 2.7.7.6 | 4 | 1 | 47.61 |
| 995 | IPR007641,IPR009674 | 4 | 2.7.7.6 | 4 | 1 | 47.61 |
| 996 | IPR007120,IPR009674 | 4 | 2.7.7.6 | 4 | 1 | 47.61 |
| 997 | IPR007075,IPR007083 | 4 | 2.7.7.6 | 4 | 1 | 47.61 |
| 998 | IPR007075,IPR007081 | 4 | 2.7.7.6 | 4 | 1 | 47.61 |
| 999 | IPR007075,IPR007080 | 4 | 2.7.7.6 | 4 | 1 | 47.61 |
| 1000 | IPR007073,IPR007083 | 4 | 2.7.7.6 | 4 | 1 | 47.61 |
| 1001 | IPR007073,IPR007081 | 4 | 2.7.7.6 | 4 | 1 | 47.61 |
| 1002 | IPR007073,IPR007080 | 4 | 2.7.7.6 | 4 | 1 | 47.61 |
| 1003 | IPR007073,IPR007075 | 4 | 2.7.7.6 | 4 | 1 | 47.61 |
| 1004 | IPR007066,IPR007075 | 4 | 2.7.7.6 | 4 | 1 | 47.61 |
| 1005 | IPR007066,IPR007073 | 4 | 2.7.7.6 | 4 | 1 | 47.61 |
| 1006 | IPR000722,IPR007075 | 4 | 2.7.7.6 | 4 | 1 | 47.61 |
| 1007 | IPR000722,IPR007073 | 4 | 2.7.7.6 | 4 | 1 | 47.61 |
| 1008 | IPR000684,IPR007083 | 4 | 2.7.7.6 | 4 | 1 | 47.61 |
| 1009 | IPR000684,IPR007081 | 4 | 2.7.7.6 | 4 | 1 | 47.61 |
| 1010 | IPR000684,IPR007080 | 4 | 2.7.7.6 | 4 | 1 | 47.61 |
| 1011 | IPR000684,IPR007075 | 4 | 2.7.7.6 | 4 | 1 | 47.61 |
| 1012 | IPR000684,IPR007073 | 4 | 2.7.7.6 | 4 | 1 | 47.61 |
| 1013 | IPR000684,IPR007066 | 4 | 2.7.7.6 | 4 | 1 | 47.61 |
| 1014 | IPR000684,IPR000722 | 4 | 2.7.7.6 | 4 | 1 | 47.61 |
| 1015 | IPR011262 | 4 | 2.7.7.6 | 4 | 1 | 47.61 |
| 1016 | IPR009674 | 4 | 2.7.7.6 | 4 | 1 | 47.61 |
| 1017 | IPR007075 | 4 | 2.7.7.6 | 4 | 1 | 47.61 |
| 1018 | IPR007073 | 4 | 2.7.7.6 | 4 | 1 | 47.61 |
| 1019 | IPR000684 | 4 | 2.7.7.6 | 4 | 1 | 47.61 |
| 1020 | IPR000268 | 4 | 2.7.7.6 | 4 | 1 | 47.61 |
| 1021 | IPR000008,IPR000719,IPR000961 | 11 | 2.7.1.- | 8 | 0.73 | 45.19 |
| 1022 | IPR000008,IPR000961 | 11 | 2.7.1.- | 8 | 0.73 | 45.19 |
| 1023 | IPR000008,IPR000719 | 11 | 2.7.1.- | 8 | 0.73 | 45.19 |
| 1026 | IPR002379 | 19 | 3.6.3.14 | 19 | 1 | 33.03 |
| 1027 | IPR000568 | 16 | 3.6.3.14 | 16 | 1 | 33.03 |
| 1028 | IPR000194,IPR000793,IPR004100 | 15 | 3.6.3.14 | 15 | 1 | 33.03 |
| 1029 | IPR000793,IPR004100 | 15 | 3.6.3.14 | 15 | 1 | 33.03 |
| 1030 | IPR000194,IPR004100 | 15 | 3.6.3.14 | 15 | 1 | 33.03 |
| 1031 | IPR000194,IPR000793 | 15 | 3.6.3.14 | 15 | 1 | 33.03 |
| 1032 | IPR004100 | 15 | 3.6.3.14 | 15 | 1 | 33.03 |
| 1033 | IPR000793 | 15 | 3.6.3.14 | 15 | 1 | 33.03 |
| 1034 | IPR000194 | 15 | 3.6.3.14 | 15 | 1 | 33.03 |
| 1035 | IPR009230 | 13 | 3.6.3.14 | 13 | 1 | 33.03 |
| 1036 | IPR008688 | 6 | 3.6.3.14 | 6 | 1 | 33.03 |
| 1037 | IPR001469 | 6 | 3.6.3.14 | 6 | 1 | 33.03 |
| 1038 | IPR000711 | 6 | 3.6.3.14 | 6 | 1 | 33.03 |
| 1039 | IPR002842 | 4 | 3.6.3.14 | 4 | 1 | 33.03 |
| 1040 | IPR002699 | 4 | 3.6.3.14 | 4 | 1 | 33.03 |
| 1043 | IPR000095,IPR000719 | 8 | 2.7.1.37 | 8 | 1 | 22.35 |
| 1044 | IPR000095 | 8 | 2.7.1.37 | 8 | 1 | 22.35 |
| 1045 | IPR000719,IPR001849 | 6 | 2.7.1.37 | 6 | 1 | 22.35 |
